# Characterization of stem cell and cancer stem cell populations in ovary and ovarian tumors

**DOI:** 10.1186/s13048-018-0439-3

**Published:** 2018-08-18

**Authors:** Seema C. Parte, Surinder K. Batra, Sham S. Kakar

**Affiliations:** 1 Department of Physiology, University of Louisville, 505 South Hancock Street, CTRB, Room 322, Louisville, 40202 KY USA; 2 James Graham Brown Cancer Center, University of Louisville, Louisville, 40202 KY USA; 3 Department of Biochemistry and Molecular Biology, University of Nebraska, Omaha, 68198 NE USA

**Keywords:** Ovarian cancer stem cells, Ovarian stem cells, Cancer stem cells, Ovarian cancer, Ovarian tumor, Ki67, High grade metastatic ovarian cancer

## Abstract

**Background:**

Ovarian cancer is a complicated malady associated with cancer stem cells (CSCs) contributing to 238,700 estimated new cases and 151,900 deaths per year, worldwide. CSCs comprise a tiny fraction of tumor-bulk responsible for cancer recurrence and eventual mortality. CSCs or tumor initiating cells are responsible for self-renewal, differentiation and proliferative potential, tumor initiation capability, its progression, drug resistance and metastatic spread. Although several biomarkers are implicated in these processes, their distribution within the ovary and association with single cell type has neither been established nor demonstrated across ovarian tumor developmental stages. Therefore, precise identification, thorough characterization and effective targeted destruction of dormant and highly proliferating potent CSC populations is an immediate need.

**Results:**

In view of this, distribution of various CSC (ALDH1/2, C-KIT, CD133, CD24 and CD44) and cell proliferation (KI67) specific markers in the ovarian surface epithelium (OSE) and cortex regions in normal ovary, and benign, borderline and high grade metastatic ovarian tumors by immuno-histochemistry and confocal microscopy was studied. We further confirmed their expression by RT-PCR analysis. Co-expression analysis of stem cell (OCT4, SSEA4) and CSC (ALDH1/2, CD44 and LGR5) markers with proliferation marker (KI67) in HG tumors revealed dual positive proliferating stem and CSCs, few non-proliferating stem/CSC (SSEA4^+^/KI67^−^ and ALDH1/2^+^/KI67^−^) and only KI67^+^ cells in cortex, signifying dynamic populations and interesting cellular hierarchy in cortex region. Smaller spherical (≤ 5 μm) and larger spindle/elliptical shaped (~ 10 μm) cell populations with high nucleo-cytoplasmic ratio were detected across all samples (including normal ovaries) but with variable distribution and characteristic stage-wise marker expression across different tumor stages.

**Conclusions:**

Diverse stem and CSC populations expressing characteristic markers revealing distinct phenotypes (spherical ≤5 μm and spindle/elliptical ~ 10 μm) were distributed within different tumor stages studied signifying dynamic and probable functional hierarchy within these cell types. Involvement of extra-ovarian sites of origin of stem and CSCs requires rigorous evaluation. Quantitative analysis of potent CSC populations, their mechanisms and pathways for self-renewal, chemo-resistance, metastatic spread etc. with respect to various markers studied, will provide better insights and targets for developing effective therapeutics to prevent metastasis and eventually help improve patient mortality.

**Electronic supplementary material:**

The online version of this article (10.1186/s13048-018-0439-3) contains supplementary material, which is available to authorized users.

## Background

While constituting only 3% of the cancer cases affecting women, ovarian cancer is a fatal malady due to its unpredictable prognosis, poor refractory outcome, onco-therapeutic resistance, metastatic spread and ultimate relapse with no signs of improvement in patient survival to date [1]. In recent years, existence of various cancer stem cells (CSCs) in ovarian tumor, as well as in ascites from patients with ovarian cancer have been reported as a cause of tumor relapse and recurrence, thus imposing a clinical problem [2–4]. Various specific markers including ALDH1/2, CD133, CD117, CD24, CD34, CD44, CD133, EpCAM, LGR5 and LY6A have been employed for isolation and characterization of ovarian CSCs from ovarian cancer cell lines, patients’ tumors and ascites [5, 6]. CSCs also known as tumor initiating/propagating cells, as per the CSC hypothesis, suggest hierarchical organization of cell subsets that possess self-renewal potential, tumor initiation capability and tumor progression [7]. Presently, it is established that tumor is comprised of heterogeneous cancer cell populations at various stages of differentiation and a tiny fraction of CSC populations. This heterogeneity leads to a serious bottleneck for effectively targeting these highly dynamic, transitioning populations of CSCs. Existence of diverse CSC populations with stem cell like characteristics at a given point and a cross talk between tissue microenvironment, various genetic and non-genetic (epigenetic) factors and dysregulation between these mechanisms impose drug resistance, tumor recurrence and metastasis [6, 8]. Combination of specific multiple markers such as ALDH^hi^/CD44^hi^/CD24^low^ (breast cancer), CD133^hi^/CD44^hi^/Nestin^hi^ (glioblastoma), CD44^hi^/Lgr5^hi^/CD133^hi^ (colon cancer) and other solid tumors have been identified (reviewed in [9]). While a stage- specific identity of ovarian CSCs is missing, their expression in normal ovary and at various stages of tumorigenesis and the marker profile for highly proliferating CSCs in metastatic stage in the context of ovarian stem cells remains unknown. Hence purpose of present study is to (i) identify stem cells residing in normal ovary versus ovarian tumors including benign (BN), borderline (BL) and high grade (HG) (malignant stage) and (ii) delineate if highly proliferating cells in HG ovarian tumor are differentiated cancer cells and/or (cancer) stem cell populations per se by using a panel of markers.

In our present study, we provide a glimpse of the diverse populations of cells with stem cell characteristics and proliferating cancer cells persisting in normal ovary and their (probable) counterparts in various stages of tumorigenesis. To the best of our knowledge, it is the first report showing/describing in detail for the existence, distribution and abundance of various stem cell populations in normal ovary and CSCs in ovarian tumors at various stages of tumorigenesis using several relevant markers, thus implicating the possibility of good (normal) stem cells going bad and ugly (as CSCs). Understanding the precise mechanisms responsible for and the factors influencing initiation of this probable cellular transformation and subtle differences between the ‘good, and the bad and ugly’ would lead us to achieve further milestones in cancer stem cell research.

## Methods

### Ethical permission for obtaining normal ovarian and tumor tissue

Tissue samples were collected from ovarian cancer patients admitted to the James Graham Brown Cancer Center, University of Louisville. Procedures followed were as per the ethical protocols approved by the Institutional Review Board (IRB). Informed patient consent was obtained prior to surgery. Of total 50 samples, 34 ovarian tissue samples were obtained from the Department of Pathology, University of Louisville after thorough examination by an experienced pathologist (*n* = 4 [NO], *n* = 10 [BN], *n* = 10 [BL] and *n* = 10 [HG]) [NO: Normal Ovary, BN: Benign tumor, BL: Borderline tumor, HG: High grade metastatic tumor]. Sixteen fresh/frozen samples (*n* = 4 for each category - NO, BN, BL and HG) were obtained from institutional bio-repository and were used for reverse transcription polymerase chain reaction (RT-PCR) analysis. All the experiments were performed in triplicates. Chemicals, reagents and plastic-ware were procured from Sigma-Aldrich or stated otherwise.

### Immunohistochemistry of ovarian tissue and tumor sections

Paraffin embedded ovarian sections from NO, BN, BL and HG were prepared by standard histology procedures. Sections were de-paraffinized in xylene. Endogenous peroxidase activity (abundant in the red blood cells) was quenched by incubating the sections in 3% hydrogen peroxide in 100% methanol for 60 min followed by rehydration in alcohol grades. Antigen retrieval and staining of each tissue section for specific stem cell/CSC marker was performed as described previously [10]. Immuno-staining pattern was assessed by scanning the slides using an Aperio Scanscope digital pathology scanner (Leica Biosystems) and Olympus IX71 inverted microscope (Olympus, Tokyo, Japan). Representative images were photographed and specific staining signals were documented.

### Immunofluorescence staining, co-localization and confocal microscopic analysis

Protocol for immunofluorescence staining and dual staining (co-localization studies) was similar to immuno-histochemistry. Endogenous peroxidase (3% H_2_O_2_) quenching step was omitted during immunofluorescence staining and intermediate washing steps were performed using PBS containing 0.5% BSA as described previously [10]. Antibody dilution for each was optimized to provide specific binding with no background as detailed in Table 1. Tissue sections were viewed using Nikon (Eclipse TI) confocal microscope and documented using NIS Elements AR software (version 4.5.1). The slides were thoroughly scanned under the confocal microscope and representative 10–12 images for each section were captured at 40X with 2.5X zoom to focus upon positive signals emitted by small 5–10 μm (cancer) stem cells of interest in each sample. Dual immuno-fluorescence protocol was identical to immuno-fluorescence protocol, except each of the two primary and secondary antibodies were applied simultaneously to each tissue section on day 1 and 2 respectively. Alexa Fluor 488 and 568-labeled anti-mouse and anti-rabbit IgG were used at 1:500–1:1000 dilution followed by counter staining with nuclear dye DAPI. The sections were examined under confocal microscope. Uniform instrument parameters were employed for confocal microscopy imaging for each antibody and on an average 7–10 images (per sample/antibody) were captured, further analysed and compiled to prepare the composite panels comprising of individual cells captured from various regions of a single field or from multiple fields that were scanned and documented.

**Table 1:** Antibodies used for identification of stem cells, cancer stem cells and proliferating cells from ovaries and ovarian tumor tissue

Antibody Name	Catalogue Number	Source/Host species	Cross Reactivity	Isotype	Locali-zation	Dilution	Source-Brand	Classification of Markers
ALDH ½	SC-166362	Mouse	Mouse and human	IgG_2b_	C,N	1/1500	Santa Cruz Biotechn-ology	CSC
CD117/C-KIT	AB-721-SAB4300489	Rabbit	Human	IgG	C	1/250	Sigma	CSC
CD133	MAB4399 Clone 17A6.1	Mouse	Human	IgG2aκ	S	1/100	EMD Millipore	CSC
CD133	NB120–16518	Rabbit	Human, Mouse, and Rat	affinity isolated	S	1/100	Novus Biologicals	CSC/HSC
CD24	SAB1402713-100UG	Mouse	Human	IgG2aκ	S	1/50	Sigma	CSC
CD44-PE/Cy5	103010	Mouse	Mouse, Human	Rat IgG2b, κ	S	1/50	BioLegend	CSC
CD44	SAB1405590	Mouse	Human	affinity isolated	S	1/50	Sigma	CSC
KI67	SAB4501880-100UG	Rabbit	Human	affinity isolated	N,C	1/50	Sigma	PrC
LGR5	MABD148 clone 5G10.1	Mouse	Human	IgG2bκ	S	1/500	Sigma	SC/CSC
OCT-4	MAB4419A4	Mouse	Human and Mouse	IgG1	N,C	1/250	Sigma	SC
SSEA-4	MAB4304	Mouse	Human and Mouse	IgG3	S	1/50	EMD Millipore	SC

*CSC* Cancer Stem Cell Marker, *HSC* Hematopoietic Stem Cell Marker, *SC* Stem Cell Marker, *PrC* Proliferation Cell Marker, *C* Cytoplasmic marker, *S* Surface marker, *N* Nuclear marker

### RNA extraction, cDNA preparation and RT-PCR

RNA from NO, BN, BL and HG tissues was prepared, purified and subjected to RT-PCR amplification using specific primers (detailed in Table 2) and conditions (described in [10]). Amplified products were analyzed on 2% agarose gels.

**Table 2:** Primer details

Gene	Primer Sequence	Annealing Temperature (°C)	Amplicon size (base pair)
GAPDH^a^	F: 5-TGATGACATCAAGAAGGTGGT-3	60	240
	R: 5-TCCTTGGAGGCCATGTGGGCC-3		
ALDH^b^	F: 5-GCACGCCAGACTTACCTGTC-3	60	91
	R: 5-CCACTCACTGAATCATGCCA-3		
C-kit^b^	F: 5-GGCATCACGGTGACTTCAAT-3	62	244
	R: 5-GGTTTGGGGAATGCTTCATA-3		
CD133^b^	F: 5-AGTGGCATCGTGCAAACCTG-3	62	184
	R: 5-CTCCGAATCCATTCGACGATAGTA-3		
CD24^b^	F: 5-TGCTCCTACCCACGCAGATT-3	60	89
	R: 5-GGCCAACCCAGAGTTGGAA-3		
CD44^b^	F: 5-CCAATGCCTTTGATGGACCA-3	60	334
	R: 5-TGTGAGTGTCCATCTGATTC-3		
Ki67^c^	F: 5-GAGGTGTGCAGAAAATCCAAA-3	60	78
	R: 5-CTGTCCCTATGACTTCTGGTTGT-3		

^a^house-keeping gene; ^b^cancer stem cell gene; ^c^cell proliferation gene, *GAPDH* Glyceraldehyde 3-phosphate dehydrogenase

## Results

**Fig. 1:**
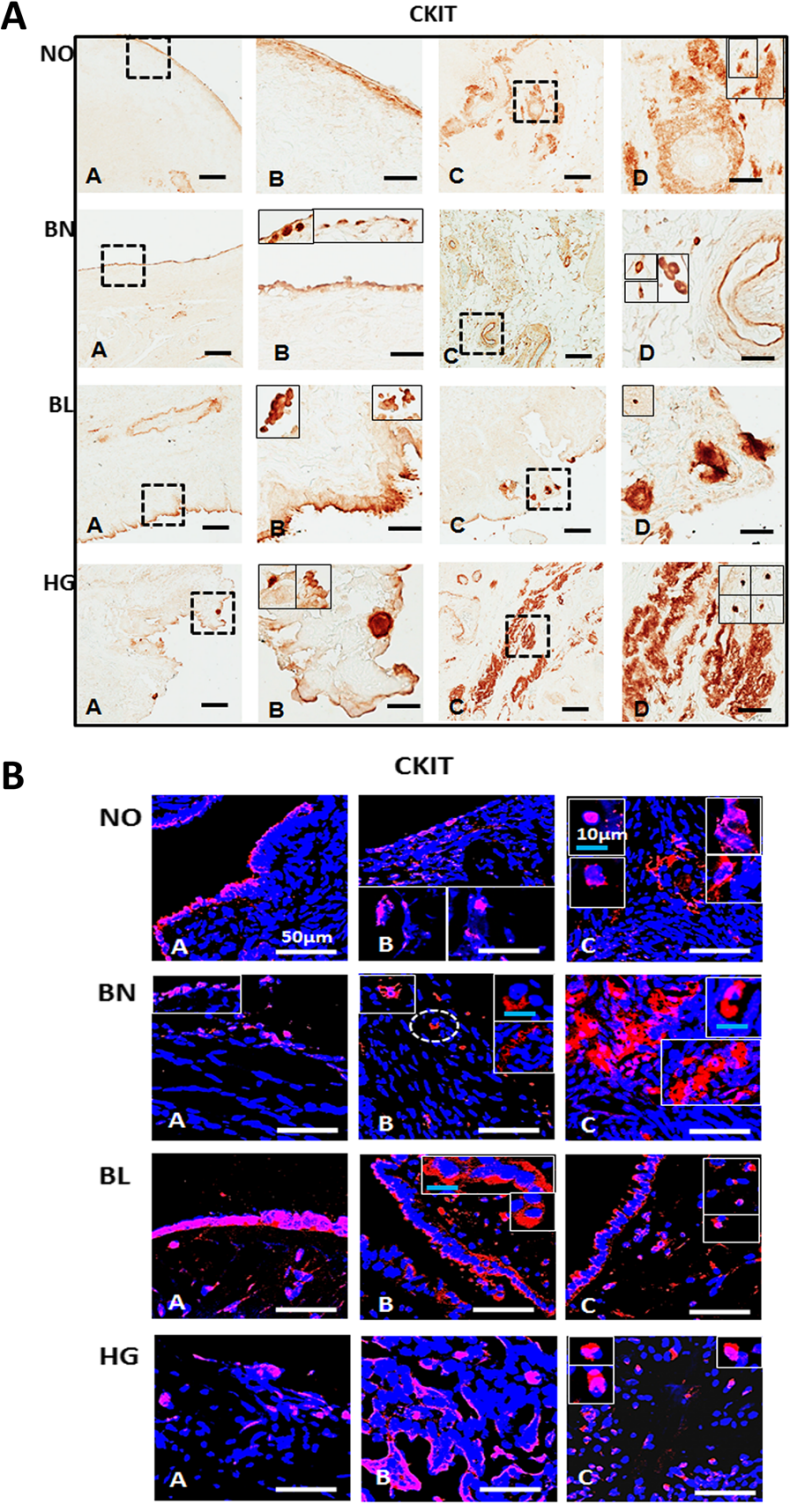
Immunolocalization of CD117/C-KIT in normal ovarian and tumor tissue sections: **[A]** Immunohistochemical detection of Anti-C-KIT polyclonal antibody in OSE (A, B) and ovarian cortex (C, D). Region between dotted boxes in A, C is magnified in B, D respectively. Diffused, faint signals were localized to OSE layer of NO and BN, BL and HG ovarian tumor tissue. C-KIT^+^ cells appear as single isolated or as clusters in cortex region across all tissue sections. More C-KIT^+^ immuno-stained cells per field were noticed in BL and HG than NO and BN. Insets denote individual spherical and elongated/spindle shaped cells at higher magnification from other fields of focus. Scale bar = 100 μm (A, C) and 25 μm (B, D) respectively. **[B]** Immunofluorescence staining of CD117/C-KIT in OSE layer (A, B) and cortex (C) both reveal specific CD117^+^ cells consistently in NO, BN as well as BL and HG ovarian cancer tissue, where these CD117^+^ cell numbers are higher in BL OSE and HG cortex per field of focus, compared to NO and BN. White scale bar = 50 μm; blue scale bar = 10 μm. Secondary antibody was conjugated with Alexa fluor 568 and sections were counterstained with nucleus specific dye DAPI

**Fig. 2:**
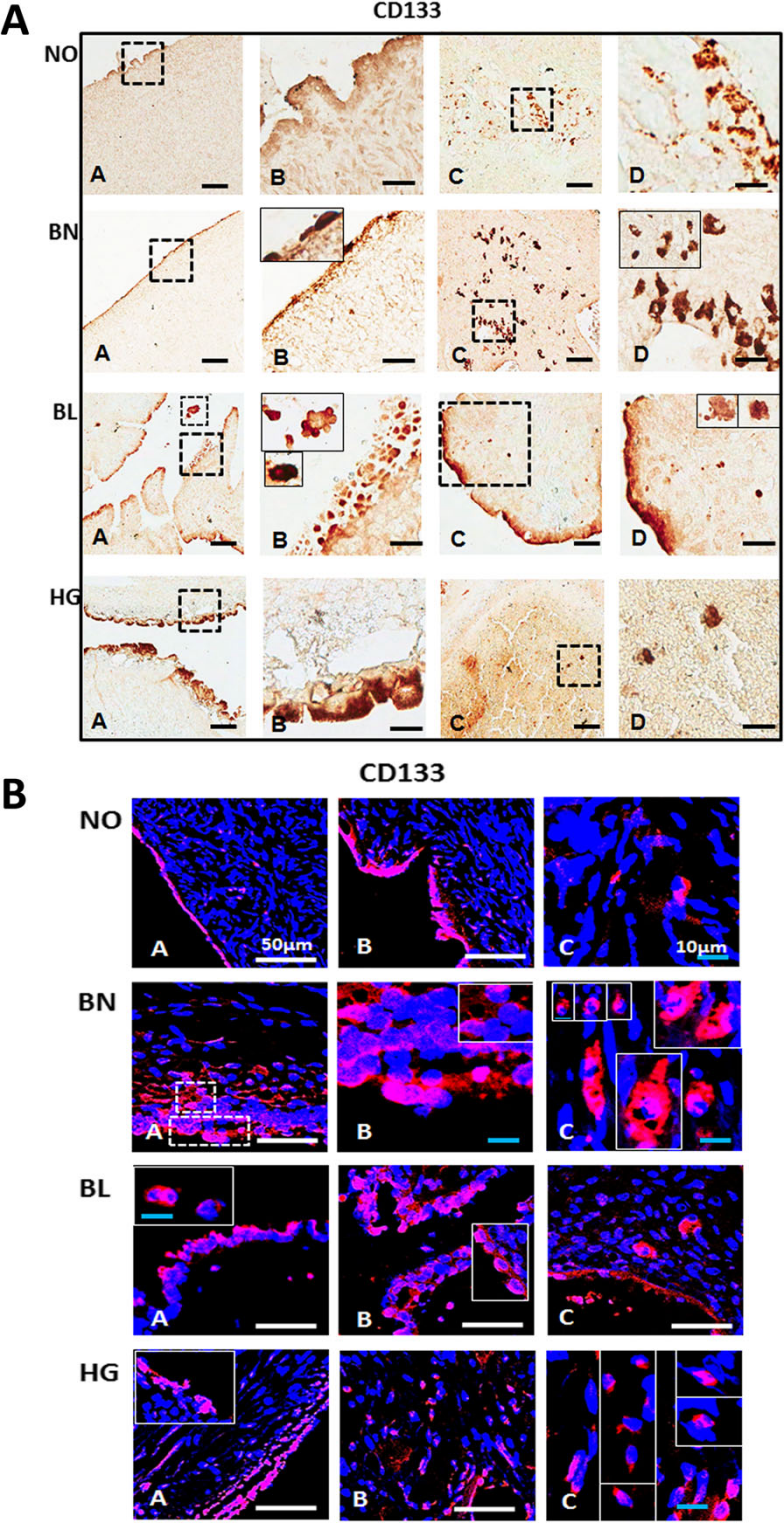
Immunostaining for CD133 in normal ovarian and tumor tissue sections: **[A]** Mouse monoclonal anti-CD133 antibody was localized in both OSE (A, B) and ovarian cortex (C, D) by immunohistochemistry. Region between dotted boxes in A, C is magnified in B, D respectively. Polar staining of CD133 is obvious in OSE layer especially in NO, BL and HG ovaries. BL ovaries exhibit multi-layered OSE. Cortex comprised of CD133^+^ cells arranged in clusters with elongated/spindle shaped morphology in NO and BN ovaries. BL ovarian cortex harbours single spherical cell clusters distributed throughout. HG comprised more of large CD133^+^ cells in OSE and few clusters in the cortex per field focussed. Insets include magnified images of cells from different fields. Scale bar = 100 μm (A, C) and 25 μm (B, D) respectively. **[B]** Immunofluorescence staining of CD133 in OSE layer (A, B) as well as cortex (C) reveals specific CD133^+^ cells with relatively higher cell numbers in BL and HG. Area within dotted lines in BN OSE (A) are magnified in (B) while elliptical/spindle shaped CD133^+^ cells in cortex from various fields were represented in the composite image in (C) of BN and HG. Large CD133^+^ cells in cortex were also observed. White scale bar = 50 μm; blue scale bar = 10 μm. Secondary antibody employed was conjugated with Alexa fluor 568 and tissue sections were counterstained with nucleus specific dye DAPI

**Fig. 3:**
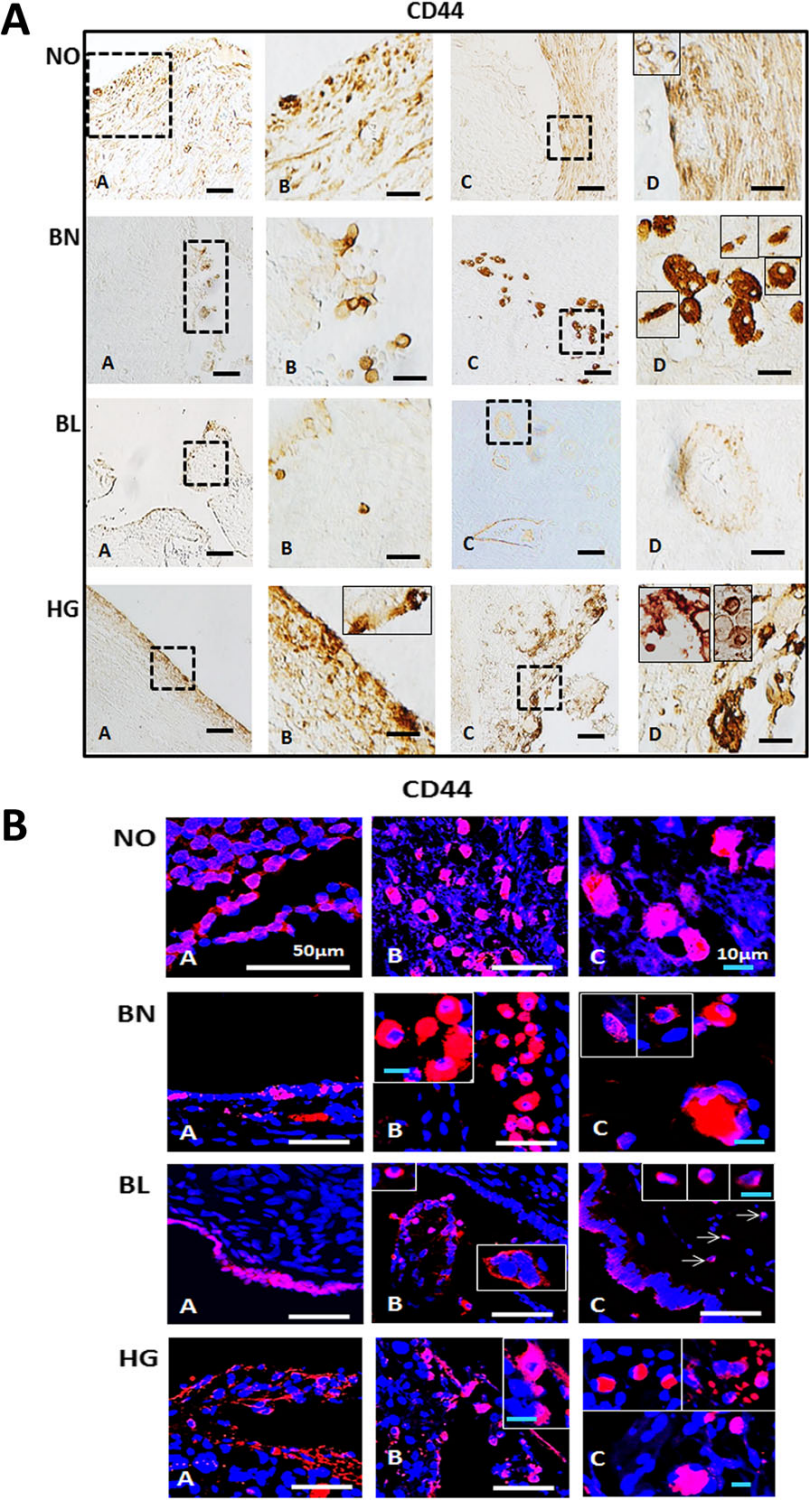
Immunolocalization of surface marker CD44 in normal ovarian and tumor tissue sections: **[A]** Monoclonal anti-CD44 antibody was localized to the OSE (A, B) and ovarian cortex (C, D) with few CD44^+^ cells visible within OSE layer in NO, BN, BL ovaries per field as compared to HG. Region between dotted boxes in A, C is magnified in B, D respectively. NO and BN ovaries appear to harbour more CD44^+^ cells in cortex as compared to BL ovaries. Some regions within the ovarian cortex possess more CD44^+^ cells adjacent to OSE layer in HG. Typically spindle/elongated shaped CD44+ cells were present in NO, BN, BL and HG cortex with round spherical cells located moreover in OSE layer. Cortex region of HG possessed both spindle and spherical shaped CD44^+^ cells. Insets in D of NO, BN, HG ovaries depict representative individual cell morphology and distribution density and localization within the cortex. Scale bar = 100 μm (A, C) and 25 μm (B, D) respectively. **[B]** Immunofluorescence staining of CD44 in OSE layer (A) and cortex (B, C) where C and insets in BN, BL and HG (B, C) represent images captured at higher magnification. BN (B) typically represents large round fluffy cells within cortex similar to those observed in HG cortex (C). CD44^+^ spindle shaped cells are found throughout all the tissue samples including NO. Single large CD44^+^ cells and multi-nucleated clusters in BN, BL (white arrows) and HG cortex were present. White scale bar = 50 μm; blue scale bar = 10 μm. Secondary antibody conjugated with Alexa fluor 568 was employed and sections were counterstained with nuclear dye DAPI

**Fig. 4:**
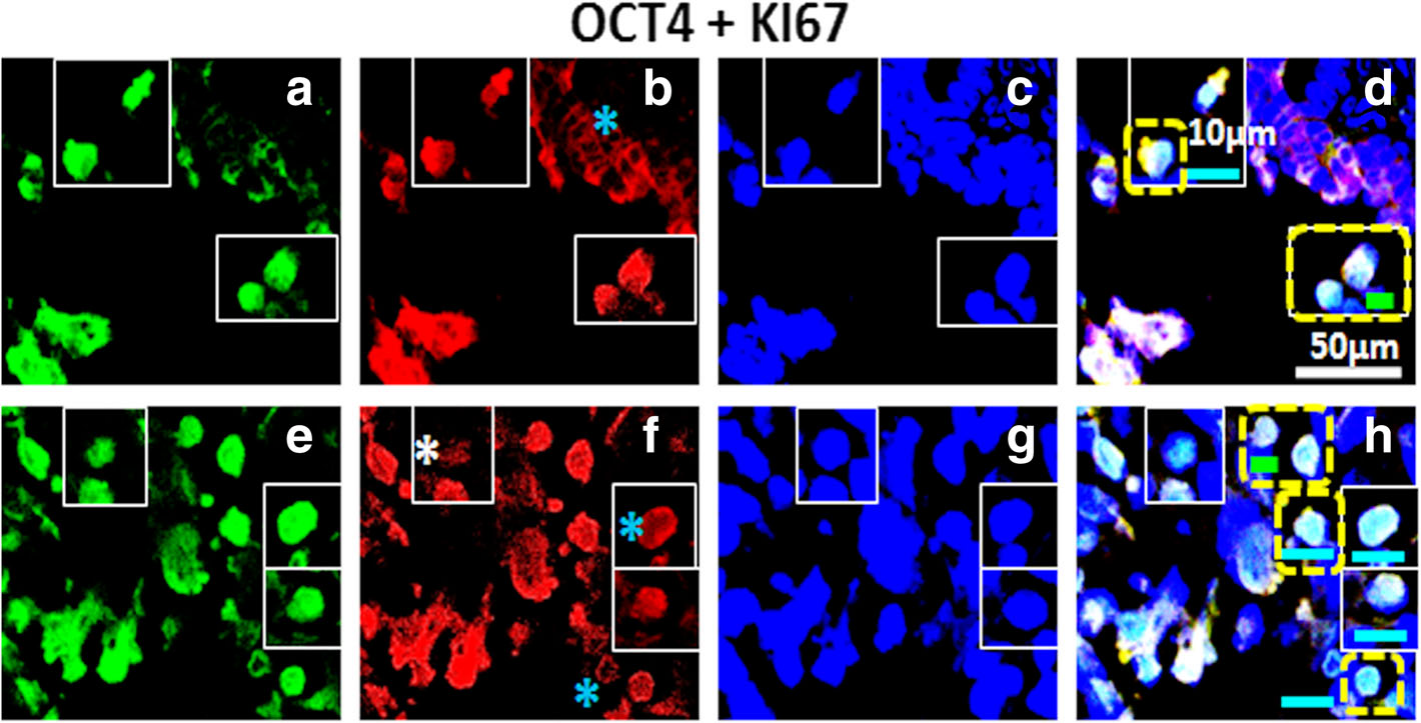
Dual labelling of High Grade (HG) ovarian tumor with OCT4 and KI67: Panel comprises of (**a**-**d**) OSE layer and (**e**-**h**) cortex [C] of which (**a**, **e**) OCT4, Alexa fluor 488, (**b**, **f**) KI67, Alexa fluor 568, (**c**, **g**) DAPI and (**d**, **h**) were merge composites. Both OSE layer (**a**-**d**) and cortex (**e**-**f**) regions of HG ovary revealed OCT4^+^/KI67^+^ cells. Cortex comprised of cells expressing nuclear OCT4^+^/KI67^+^ and cytoplasmic KI67^+^ (blue asterisk). Few OCT4^+^/KI67^−^ cells (white asterisk) were also observed in the cortex. Tiny spherical (VSELs-like) stem cells were indicated by yellow dotted square. Blue asterisk in (**f**) denotes cytoplasmic KI67. White scale bar = 50 μm, blue scale bar = 10 μm, green scale bar = 5 μm

**Fig. 5:**
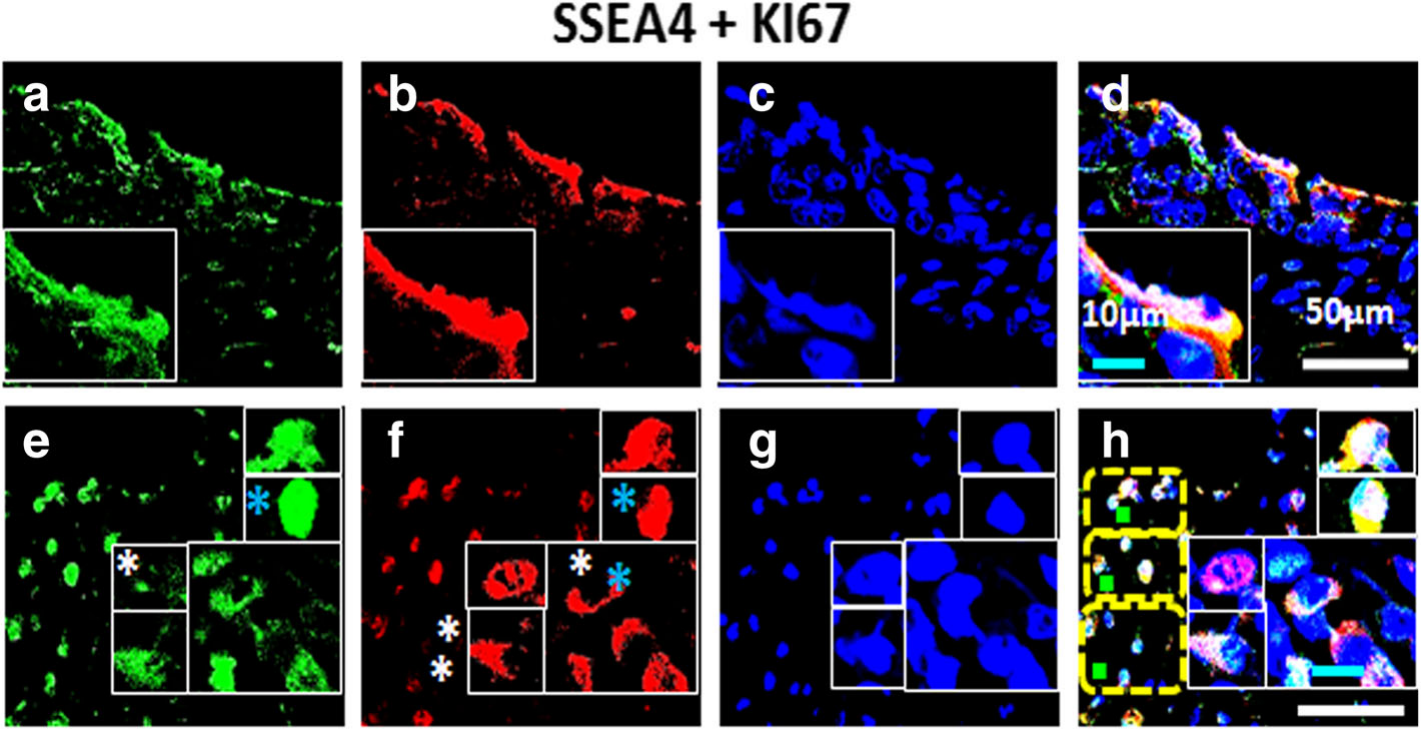
Co-localization of SSEA4 and KI67 in High Grade (HG) ovarian tumor: Panel comprises of (**a**-**d**) OSE layer and (**e**-**h**) cortex [C] of which (**a**, **e**) SSEA4, Alexa fluor 488, (**b**, **f**) KI67, Alexa fluor 568, (**c**, **g**) DAPI and (**d**, **h**) were merge composites. OSE layer prominently showed SSEA4^+^ cells that were KI67^+^ (nuclear and cytoplasmic). In some fields SSEA4^−^/KI67^+^ cells were also observed in cortex. Cytoplasmic SSEA4^+^/KI67^+^ were also observed in some fields of focus. Insets denote representative cells from same field of focus compiled in one panel. Tiny spherical (VSELs-like) stem cells were indicated by yellow dotted square. Blue asterisk denotes cytoplasmic KI67. White scale bar = 50 μm, blue scale bar = 10 μm, green scale bar=5μm (H)

**Fig. 6:**
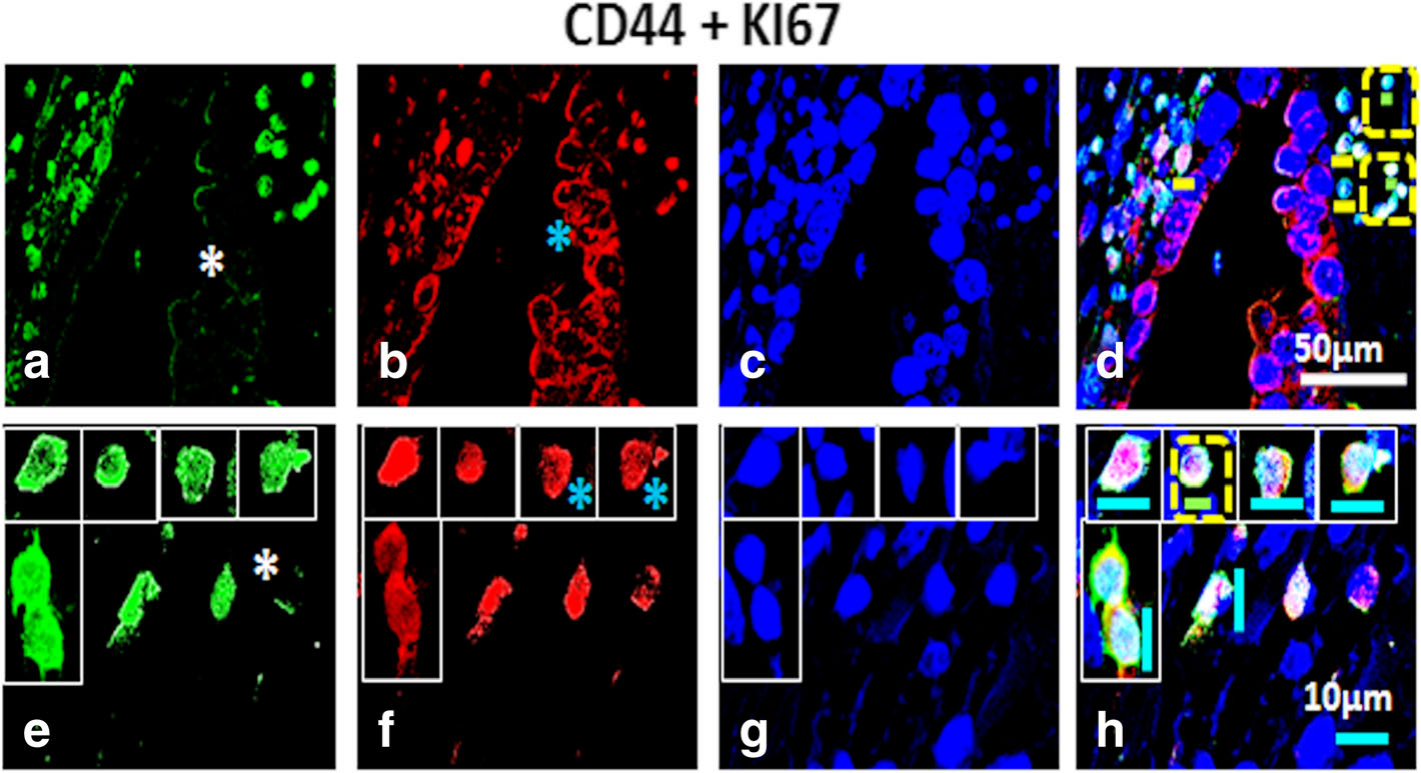
Dual labelling of High Grade (HG) ovarian tumor with CD44 and KI67: Panel comprises of (**a**-**d**) OSE layer and (**e**-**h**) cortex [C] of which (**a**, **e**) CD44, Alexa fluor 488, (**b**, **f**) KI67, Alexa fluor 568, (**c**, **g**) DAPI and (**d**, **h**) were merge composites. CD44^+^ cells situated just below OSE layer expressed KI67. Some KI67^+^ cells were prominently visible in cortex. Nuclear as well as cytoplasmic KI67^+^ cells were visible in cortex. White asterisk = CD44^−^ cells, Blue asterisk denotes cytoplasmic KI67. Tiny spherical (VSELs-like) stem cells were indicated by yellow dotted square. White scale bar = 50 μm, blue scale bar = 10 μm, yellow scale bar = 10 μm (D) and green scale bar = 5 μm (D, H)

**Fig. 7:**
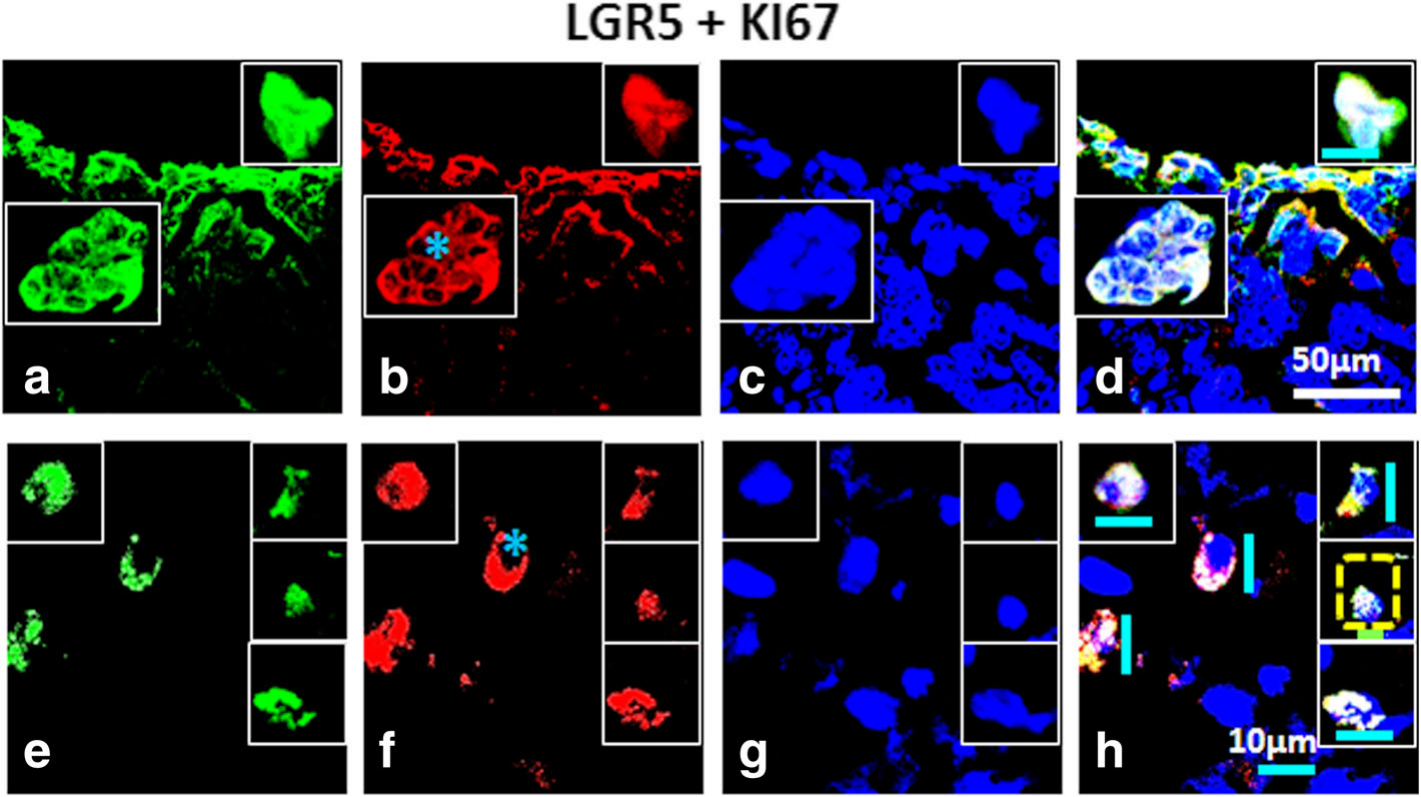
Co-expression of High Grade (HG) ovarian tumor for LGR5 and KI67: Panel comprises of (**a**-**d**) OSE layer and (**e**-**h**) cortex [C] of which (**a**, **e**) LGR5, Alexa fluor 488, (**b**, **f**) KI67, Alexa fluor 568, (**c**, **g**) DAPI and D, H) were merge composites. OSE layer and few clusters in some fields expressed both i.e. LGR5^+^/KI67^+^. Membrane bound LGR5^+^ and cytoplasmic KI67^+^ were observed within the cortex. Inset reveals cells from different fields captured at high magnification. Tiny spherical (VSELs-like) stem cells were indicated by yellow dotted square. Blue asterisk denotes cytoplasmic KI67. White scale bar = 50 μm, blue scale bar = 10 μm, green scale bar = 5 μm (H)

**Fig. 8:**
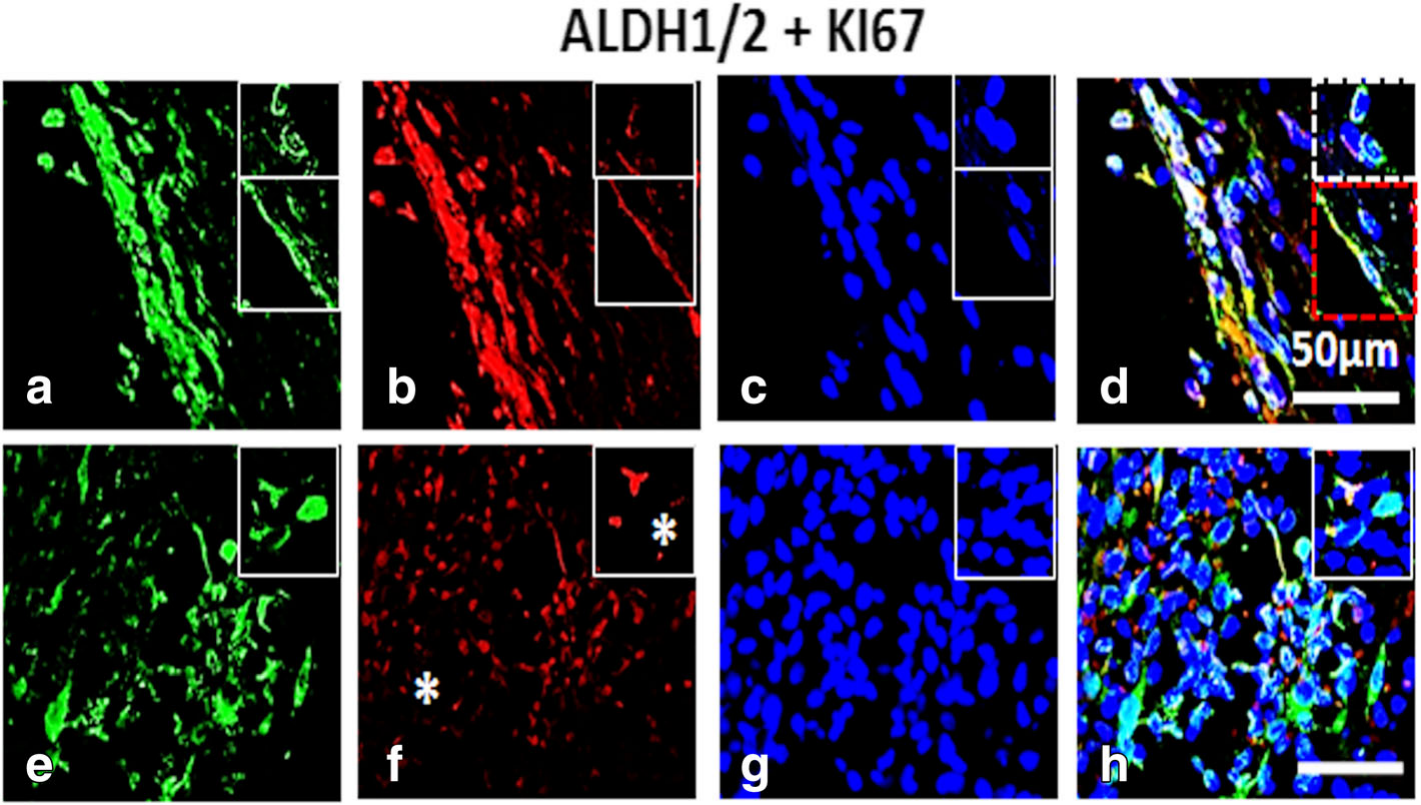
Dual labelling of High Grade (HG) ovarian tumor with ALDH1/2 and KI67: Panel comprises of (**a**-**d**) OSE layer and (**e**-**h**) cortex [C] of which (**a**, **e**) ALDH1/2, Alexa fluor 488 (**b**, **f**) KI67, Alexa fluor 568 (**c**, **g**) DAPI and (**d**, **h**) were merge composites. OSE layer revealed presence of ALDH1/2^+^/KI67^+^ cells. Some spindle shaped ALDH1/2^+^ (inset) were KI67+. Many cells observed within the cortex were ALDH1/2^+^/KI67^−^. White dotted square denotes disseminated OSE cells. Red dotted square denotes spindle shaped ALDH1/2^+^ OSE cells. KI67^−^ cells = white asterisk. White scale bar = 50 μm

In our present study, we focused on understanding the distribution of various CSC-specific markers in normal ovary and various stages of ovarian tumorigenesis (BN, BL and HG), and characterization of CSCs in HG metastatic ovarian cancer tissues (Figs. 1A, B, 2A, B and 3A, B, Additional file 1: Figure S1 and Additional file 2: Figure S2) and their co-localization with cell proliferation marker [KI67] (Figs. 4, 5, 6, 7 and 8, Additional file 3: Figure S3, Tables 3 and 4). Expression pattern of each marker within individual cells/clusters in both OSE and cortex regions in normal ovary and at various stages of tumorigenesis was analysed (Additional file 4: Table S1 and Additional file 5: Table S2). Additional positive stained cells from different regions of same or other fields of focus revealing diverse cell staining, with variable phenotype (size [5–10 μm], shape [spherical and spindle/fibroblast], localization [OSE/cortex]) and expression of various combination of markers were included in insets throughout. Localization of each CSC-marker is provided below:

**Table 3:** Details of markers and techniques used in the study

Markers/Genes	IHC	IF	RT-PCR
OCT-4^a^	–	√	–
SSEA-4^a^	–	√	–
ALDH1/2^b^	–	√	√
LGR5^b^	–	√	–
CD117/C-KIT^b^	√	√	√
CD133^b^	√	√	√
CD24^b^	√	–	√
CD44^b^	√	√	√
KI67^p^	√	√	√
GAPDH^hk^	–	–	√

*IHC* Immunohistochemistry, *IF* Immunofluorescence; ^a^Stem cell marker; ^b^Cancer stem cell marker, ^p^ Proliferation marker, ^hk^ Housekeeping gene

**Table 4:** Expression of stem/cancer stem cell (OCT4, SSEA-4, CD44, LGR5, ALDH) and proliferation (KI67) markers after dual labeling of ovarian tissue and tumors

Markers/Genes	HG-OSE	HG-Cortex
OCT4^+^/KI67^+^	O^+^_(n)_/K^+^_(c)_, O^+^_(n)_/K^+^_(n)_	O^+^_(n)_/K^+^_(n)_, O^+^/K^−^
SSEA-4^+^/KI67^+^	S4^+^/K^+^_(n)_	S4^−^/K^+^_(n)_, S4^+^/K^−^, S4^+^/K^+^_(c)_
Inference: Stem cell and proliferation marker both are being co-expressed in OSE cells and cortex region (O^+^/K^+^ and S4^+^/K^+^). Few non-proliferating (KI67^−^) OCT4^+^ cells were also observed in the cortex region. KI67 was expressed in active phases of cell cycle [G _(1)_, S, G _(2)_], and mitosis) among the non-stem cancer/tumor cells as well as in CSCs. Majority of the cells expressing OCT4 in cortex region were also in proliferation state [O^+^_(n)_/K^+^_(n)_, O^+^_(n)_/K^+^_(c)_]. Similarly OSE and cortical cells also expressed surface/cytoplasmic (SSEA-4) and proliferation (KI67) markers [S4^+^/K^+^_(n)_, S4^+^/K^+^_(c)_]. Some cells in cortex were active in cell cycle but failed to express stem cell characteristics signifying probable non-stem cancer cells within the tumor. Some KI67^−^ cells with stem cell properties were observed [S4^+^/K^−^] thus indicating their quiescent/non-proliferating state of cell cycle/dormancy [S4^+^(c) /K^+^(c), S4^+^(c) /K^+^(n)]. KI67(c) cells are typically observed in cancer tissue and by virtue of expression of OCT4^+^ [O^+^_(n)_/K^+^_(n),_ O^+^_(n)_/K^+^_(c)_] and SSEA-4^+^ [S4^+^/K^+^_(c)_] stem cell properties signify a proliferating population of stem cells with probable tumorigenic potential.		
CD44^+^/KI67^+^	C44^+^/K^+^_(c)/(m)_	C44^+^/K^+^_(n)_, C44^+^/K^+^_(c)_, C44^−^/K^+^_(m)_
LGR5^+^/KI67^+^	L5^+^/K^+^_(c), (m)_ (OSE cells and clusters)	L5^+^/K^+^_(m)_
ALDH^+^/KI67^+^	A^+^/K^+^_(c)_ spindle shaped cells)	A^+^/K^−^, A^+^/K^+^_(c)/(m)_
Inference: Co-expression of cancer stem cell (CD44, LGR5, ALDH) and proliferation (KI67) markers in the OSE layer and cortex regions both (C44^+^/K^+^, L5^+^/K^+^, ALDH^+^/K^+^) denote prevalence of very potent populations/sub-populations of actively proliferating CSCs. LGR5 expression alone also indicates proliferating stem cells. Therefore L5^+^/K^−^ cell types were not reported. However differential expression of dual markers in both ovarian regions signifies various stages of differentiation and proliferation status of CSCs. KI67_(c), (m)_ expression along with diverse cancer stem cell characteristics [C44^+^/K^+^_(c)_, C44^−^/K^+^_(m)_, L5^+^/K^+^_(m),_ A^+^/K^+^_(c)_] reflects existence of dynamic populations of proliferating CSCs indeed.		

+: positive expression; −: negative/nil expression

O: OCT4, S4: SSEA4, K: KI67, C44: CD44, L5: LGR5, A: ALDH1/2;

(c): cytoplasmic localization; (m): membrane localization; (n): nuclear localization

### Immuno-histochemical analysis of CSC markers in normal ovary and tumor tissues

A characteristic but unique distribution pattern for each marker namely C-KIT/CD117, CD133, CD44, CD24 and ALDH1/2 (Figs. 1A, 2A and 3A, Additional file 1: Figure S1, Additional file 3: Figure S3) within OSE layer and cortex regions both were noticed (detailed in Additional file 4: Table S1).

#### C-KIT/CD117

C-KIT/CD117 is an oncogene (a tyrosine kinase receptor) and is significantly over expressed in malignant ovarian tumors and associated with poor patient survival and worse outcome [11–13]. It is expressed in fibroblast-like stromal cells (possibly of MSC origin) in ovarian carcinoma [14] in addition to normal OSE [15], thus making it imperative to further assess its expression in various CSC subsets and decipher its precise functional role in tumorigenesis. In our studies, we observed that C-KIT/CD117 was localized in OSE layer in NO, BN, BL and HG. CD117^+^ cells per field were found to be higher in BL and HG compared to NO and BN. In addition, large CD117^+^ cell clusters (tumor like) were visible in BL and HG. In contrast, NO and BN showed CD117 expression as single cells. Larger cells with bright and specific positive signals in the layer beneath OSE were visible in BN, while multiple cells in cluster detached near OSE (multi) layer in BL with specific foci. We observed CD117^+^ specific signals with bright staining spread across the cortex. Specific single or multiple cells forming a non-uniform pattern were more prominent in BL and HG compared to NO and BN (Fig. 1A), merely suggesting their putative role in ovarian tumorigenesis.

#### CD133

CD133/Prominin is a five-trans-membrane domain glycoprotein with potential role in organization of plasma membrane. CD133^+^ cells possess high clonogenic capability and are implicated in tumor development and chemo-resistance [16–19]. High levels of CD133 expression have been reported in primary ovarian tumor compared to NO and BN [20]. Surprisingly, lower levels of expression in metastatic HG have been reported [21]. In our studies, using the CD133-specific antibody, we observed the presence of bright and uniformly distributed CD133^+^ cells prominently in OSE layer throughout NO, BN, BL and HG. However, multiple clusters of CD133^+^ cells were visible in OSE layer of both BL and HG. Single or multiple CD133^+^ cells were distributed throughout the cortex in NO, BN, whereas, sparse and isolated CD133^+^ cells were detected in BL and HG cortex (Fig. 2A).

#### CD44

CD44 is expressed in lymphoid epithelial cells as well as implicated in various cellular processes such as cell-cell interaction, apoptosis, migration, invasion, tumorigenesis and metastasis. Owing to these properties, CD44 serves as effective tool for pathological diagnosis and prognostic prediction of ovarian cancer in a clinical setup. [22, 23]. It remains unknown if there exists a relationship between CD44 expression and various tumor stages. Our studies revealed presence of CD44^+^ cells in OSE layer and beneath it in NO and HG. Specific single isolated CD44^+^ cells were visible in OSE of BN and BL. Analysis of cortex revealed existence of highly specific CD44^+^ signals in either single or multiple cells (spherical and elliptical) in HG in single field of focus. Larger fluffy appearing CD44^+^ cells in BN cortex were also noticed (Fig. 3A).

#### CD24

It is a sialo-glycoprotein expressed by mature granulocytes, B cells and regulates their growth and differentiation. High levels of CD24 expression and its association with quiescence, chemo-resistance, tumor initiation, and metastatis have been reported in several cancers [24, 25]. Despite its absence in NO OSE and high expression in ovarian carcinoma, it is speculated as a non-specific ovarian tumor marker [26]. In recent studies by Korkolopoulou et al. [27] reported CD24 as a key molecule in metastasis and EMT process in tumor tissues and Caov3 cell line, suggesting its role in dissemination of ovarian CSCs to peritoneal cavity; therefore, it may serve as a potential therapeutic target. However, none of the studies correlated it with ovarian stem cells in context to localization of CD24^+^ cells in OSE or cortex region. Therefore, to define the distribution of CD24^+^ cells in normal ovary and at different stages of ovarian tumorigenesis, we analysed its expression. CD24^+^ cells were visible in OSE layer of BN, BL and HG, while no signal was detected in NO. NO, BL and HG cortex specially revealed small regions/foci with prominent positive signals while the surrounding region showed undetectable signals confirming the staining specificity. Single - spherical, and multiple-spindle and elliptical shaped CD24^+^ cells were observed (Additional file 1: Figure S1).

#### KI67

Although, various studies demonstrate correlation of KI67 expression with shorter disease free survival as well as overall patient survival (OS) [28–32], discrepancy among different studies prevail (discussed in [32]). Disputing authenticity of KI67 as a prognostic marker is attributed to onco-therapy regimen among different patients, patient status (chemo-naive versus chemo-treated), variable staining procedures adopted, technical and methodology related variations such as antibodies, specimen type and fixative type used; antigen retrieval procedure and scoring methods employed by different investigators [29, 33]. Therefore, a detailed study to define its role and significance in tumorigenesis is required.

In our study, we observed diffused staining and existence of rare KI67^+^ cells in NO tissues, while highly specific KI67^+^ cells within OSE layer in BN, BL and HG as single, isolated-spherical while spindle shaped multiple-clustered or duplet cells were observed within the cortex (Additional file 3: Figure S3). Variable shaped nuclear and cytoplasmic/membrane bound KI67^+^ signals in HG OSE and cortex revealed numerous proliferating cells (cancer cells and/or CSCs). Overall small spherical cells (~ 5 μm, which resemble VSELs [34] and larger spindle shaped (~ 10 μm) cells were prominently observed, revealing multiple CSC specific markers, suggesting dynamic hierarchy persisting similar to distinct populations described earlier in NO [35–37].

### Immunofluorescence staining of CSC markers in normal ovary and ovarian tumor

To confirm our results from immuno-histochemical analysis and to define the sub-cellular (membrane, cytoplasmic and nuclear) localization, we performed immuno-fluorescence analysis using specific antibody for each of the marker followed by high resolution confocal microscopy. Our analysis revealed the existence of small, spherical (~ 5 μm size) cells as well as large elliptical (~ 10 μm size) cells and multiple cell clusters in both OSE layer and cortex in NO and BN, BL and HG tumors (Figs. 1B, 2B, 3B, Additional file 2: Figure S2, Figs. 4, 5, 6, 7 and 8). Distribution pattern of various markers is described below and detailed in Additional file 5: Table S2.

#### C-KIT/CD117

C-KIT^+^/CD117^+^ cells were found to be localized in OSE layer of NO, BN, BL and HG. Maximal stain signals were detected per field of focus in multi-layered OSE in BL. Very specific and bright prominent cell membrane bound signals in single isolated cells within cortex and layers of cells beneath OSE were detected in NO, BN, BL and HG. The 5–10 μm size cells were detected as single entities or within multiple cell clusters in both OSE and cortex. CD117^+^ cells larger than 10 μm were typically detected in BN cortex (Fig. 1B). These results are consistent with immuno-histochemical staining reported above (Fig. 1A).

#### CD133

Single and multi-layered CD133^+^ cells in OSE of NO, BN, BL and HG tissues were clearly visible with prominent cell membrane bound CD133^+^ staining. Cells with size of 5–10 μm were clearly identified. Isolated but multiple CD133^+^ cells were visible in cortex of NO, BN and BL tumors with higher number of CD133^+^ cells/field of focus in HG (Fig. 2B). These results are consistent with immuno-histochemical staining reported above (Fig. 2A).

#### CD44

Multiple CD44^+^ cell membrane bound signals in NO, BN single isolated cells and cell clusters were visible especially in BL and HG OSE layers. Cortex also revealed single and multiple cells in proximity in specific regions. Multi-nucleated cell clusters were obvious in cortex of NO, BN and HG, while single isolated (≤10 μm) cells predominated in BL cortex. Large spherical fluffy appearing cells (either single or multi-nucleated) were typically observed in BN cortex (Fig. 3B). These results are consistent with immuno-histochemical staining reported above (Fig. 3A).

#### ALDH1/2

Single/multi-layered OSE cells revealed ALDH1/2^+^ cells with spherical and elliptical morphology in NO and BN. BL and HG OSE revealed spherical and elliptical shaped cells in clusters in addition to single isolated cells. Numerous ALDH1/2^+^ cells were observed in HG cortex as compared to NO, BN and BL cortex (Additional file 2: Figure S2). Most cells exhibited cytoplasmic signals while some cells specially noticed in NO and HG cortex revealed nuclear ALDH1/2^+^ signals. Distribution of ALDH1/2^+^ CSCs implicated in tumor progression and metastasis was recently shown by immunohistochemistry in ovarian tumors and in vitro in context to antitumor effect of novel drugs targeting CSCs [38]. Negative controls for mouse and rabbit antibodies were employed by omitting primary antibody (Additional file 6: Figure S4).

A detailed analysis of cellular phenotype and further association of cells reported above with known ovarian germline stem cells is prerogative. Thus characteristic distribution pattern of various markers signifies specific CSC populations distinctly distributed within the tumor tissues with an aim to probably perform specific functional roles in tumor progression and metastasis is speculated. This may form the basis for further analysis of each sub-population in terms of their mechanisms adopted, their functional roles and significance in the process of tumorigenesis and metastatic spread. This study in turn may aid the identification of effective therapeutic targets.

### Co-expression of stem and CSC specific markers with proliferation marker in high grade ovarian tumor tissue sections

It is vital to understand cellular identity of proliferating cell fraction in HG tumors. Since CSCs have been reported to play critical role in metastasis, therefore, to comprehend the phenomenon of metastasis and ascites formation, we performed co-expression of KI67 with stem cell and CSC specific markers. In our study, we observed that pluripotency related stem cell marker OCT4 was predominantly expressed in both OSE layer (Fig. 4a-d) and cortex (Fig. 4e-h). In both locations, OCT4^+^ cells also expressed proliferation marker KI67 with mostly nuclear signals for both markers while few cells with cytoplasmic KI67 signals (blue asterisk) were detected. Expression of both OCT4 and KI67 was found to be variable. A few OCT4^+^ cells expressed low/undetectable levels of KI67 (white asterisk), possibly signifying different stages of differentiation of CSCs. Other surface marker typical of pluripotent stem cells that is SSEA4 (Fig. 5) was found to be expressed in both OSE layer (Fig. 5a-d) and cortex (Fig. 5e-h) of HG. Some of the SSEA4^+^ cells in cortex showed co-expression of KI67. On the other hand some cells showed only expression of KI67 (were SSEA4^−^ indicated by white asterisk). Cortex region of normal ovary revealed more of these dynamically mixed (SSEA4^+^/KI67^+^, SSEA4^−^/KI67^+^, SSEA4^+^/KI67^−^) cell populations and revealed cytoplasmic signals (blue asterisk) similar to previous report in NO [36, 39].

CD44 (CSC-surface marker) positive cells showed co-expression of KI67. These cells revealed nuclear as well as cytoplasmic and membrane bound (blue asterisk) KI67 signals in cortex. A large number of bright dual positive cells were detected in the cortex region (Fig. 6). Some of the KI67^+^ cells revealed undetectable CD44 staining (white asterisk), suggesting variable expression of CD44 marker at various stages of proliferation and differentiation of cancer cells as well as CSCs. Similarly LGR5 (a surface G-protein coupled receptor) revealed its presence in OSE layer as well as cortex region of HG tumor. LGR5^+^ cells in OSE layer exhibited membrane localization, whereas, KI67^+^ signals revealed both nuclear as well as cytoplasmic localization (blue asterisk). Cell clusters positive for expression of both markers (LGR5^+^/KI67^+^) were found in OSE layer as well as in cortex region suggesting proliferating CSCs.

ALDH1/2^+^/KI67^+^ cells were mainly localized in OSE layer. Many spindle shaped KI67^−^ cells (white asterisk) expressing ALDH1/2 were observed in the cortex (Fig. 8), probably suggesting non-proliferating CSC population within the cortex. However further in-depth study to confirm this hypothesis is required. Negative controls for mouse and rabbit antibodies were employed by omitting primary antibody for both immuno-staining methods (Additional file 6: Figure S4).

### RT- PCR amplification of stem cell, CSC specific transcripts and proliferation marker in normal ovarian and tumor tissues

**Fig. 9:**
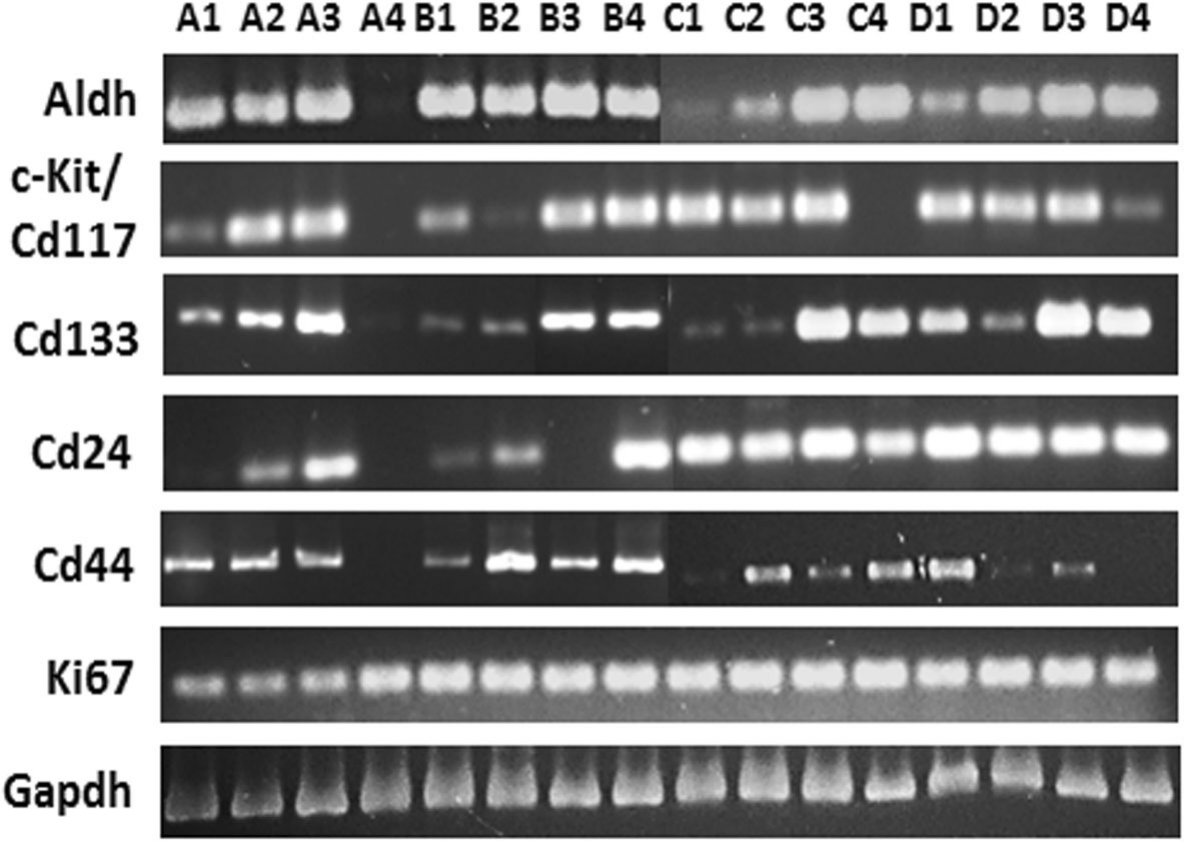
RT-PCR and mRNA transcript analysis for cancer stem cell and proliferation markers in normal ovarian and tumor tissue: Expression pattern of RT-PCR amplicons for cancer stem cell related genes (*Aldh, Ckit, Cd133, Cd24, Cd44*) and proliferation marker (*Ki67*) and housekeeping gene (*Gapdh*) were detected in various normal human ovarian and tumor tissue samples. *1 = NO, 2 = BN, 3 = BL, 4 = HG; A, B, C, D = set 1–4 of patient samples. NO = normal ovarian tissue, BN = benign, BL = borderline, HG = high grade ovarian tumor*

To confirm for the existence of various CSC markers in ovarian tissues at various stages of ovarian tumorigenesis, we performed RT-PCR amplification of each marker gene using specific primers. As shown in Fig. 9, amplified gene products of expected size for CSC and proliferation specific markers were detected in most samples analysed. We used four normal ovarian (NO) tissues and four sets of patient tissues belonging to BN, BL, and HG tumors. RT-PCR analysis without cDNA template served as a negative control. RT-PCR analysis presents a semi-quantitative measure of mRNA transcript copy number expressed across various tissues. RT-PCR analysis of normal and tumor tissues confirmed the expression observed by both immuno-histochemical and immuno-fluorescence methods for each marker. As expected, some variation in the expression of each marker gene was observed among patients, but tumor stage-specific differences in expression of marker genes was not obvious due to immense cell heterogeneity within tumor tissues and lack of RT-PCR analysis on enriched cell populations per se (Fig. 9).

## Discussion

The CSC theory put forth initially by Virchow and Cohnheim hypothesized CSCs as “dormant embryonic tissue remnants”, which later was replaced by the concept of “tissue specific stem cells as cancer cell origin” [40]. The adult human OSE is described as a germinal layer [41], and is implicated in possessing primitive germ cells/precursor cells and their differentiation into germline cells. Furthermore, both quiescent and actively cycling stem cell populations are reported to exist in adult tissues such as bone marrow, skin, intestinal epithelium and hair follicle [42]. Several investigators have generated significant knowledge about germline stem cells in normal physiology and CSCs in tumors per se, however, several aspects in ovarian CSCs remain unknown to date. These include: i) whether CSCs are harboured within surface epithelium lining of the ovary, ii) they exist in the interior stroma/cortex region as well, iii) whether they migrate from the germinal layer to the interior, and iv) whether there is independent or simultaneous infiltration of stem cells from neighbouring reproductive organs (i.e. extra ovarian sites). An unequivocal opinion regarding the true cell of origin for epithelial ovarian cancer fails to exist because the OSE layer and oviductal fimbriae both share a common origin of transitional epithelium with incomplete commitment making these zones susceptible to neoplastic transformation [43]. While extra-ovarian source i.e. oviductal epithelium or fallopian tube fimbrial cells [44, 45] were suggested in human ovarian cancer; at the same time hilum region of mouse ovary, the transitional (or junction) area between OSE, mesothelium and tubal (oviductal) epithelium were proposed as the putative stem cell niche with implications in malignant transformation [46]. Lack of common histological parameters between normal ovary and tumors and difficulty to identify reproducible precursor lesions [45] in the ovary instigate further comprehensive research in this direction.

Present study and our recent publication [10] suggest for the existence of stem cell populations with CSC specific characteristics and many proliferating cells, alongside subsets of non-proliferating (probably dormant) stem cells (i.e. KI67^−^) expressing stem and CSC profile. Intriguingly stem cell subsets/compartments in normal ovary shared CSC specific markers expressed in tumors; while tumors possessed stem cell specific markers, thus implicating probable involvement of normal ovarian stem cells in the transformation and initiation of cancer (as proposed recently by Kenda-Suster et al. [47] in serous ovarian carcinoma). On similar lines in our recent study, we reported existence of differentially expressed germ line (stem cell) markers in CSCs (ovarian tumor) and stem cells (normal ovary) [10]. Other past studies suggest that OSE and cortex from normal adult ovarian tissues possess (OCT4^+^, SSEA4^+^) tiny, spherical, stem cells and committed progenitor populations [36, 39, 48] which undergo asymmetric cell division [49] and upon proliferation form germ cell nests/clusters [39, 49]. Similar cell clusters were observed in our recent [10] and current study possibly indicating proliferating stem cells persisting not only in normal ovary but present in ovarian tumor OSE and probably implicated in tumorigenesis as recently suggested by Virant Klun and group [50, 51].

### CSC heterogeneity

In present study, CSC sub-populations were detected by immuno-staining for CSC specific markers and co-expression analysis of same with proliferation marker KI67 to analyse the distribution, abundance and heterogeneity of persisting cell types within OSE and cortex regions of both normal ovary and ovarian cancer tissues at various stages of tumorigenesis. We did not investigate tumor progression from a particular stage to the next and associated changes in terms of CSC profile, but employed archival and fresh patient samples surgically excised at the Cancer Center, meeting the criteria of representing various stages of tumor development (BN, BL, HG) besides normal sample (NO) for immuno-staining and RT-PCR studies. We investigated by RT- PCR the presence of transcripts for each marker using specific primers. Immuno-staining of each marker (Figs. 1, 2 and 3, Additional file 1: Figure S1, Additional file 2: Figure S2 and Additional file 3: Figure S3) with respect to differential marker expression, distribution of signals and co-localization with proliferation marker KI67, detected heterogeneous sub-populations of cells (Figs. 4, 5, 6, 7 and 8). This scenario correlates to certain extent (if not completely) and possibly justifies the variation in expression of various markers observed by RT- PCR (Fig. 9). A variable level of expression for each marker gene was observed in patients’ tumor tissues, which could be the result of presence of heterogeneous mixture of tumor cells in various stages of differentiation along with a tiny fraction of CSCs. Such variability in expression of each CSC marker, distinct phenotype, heterogeneity in stemness status etc. have been recently reviewed in ovarian CSCs [52]. On similar lines the findings by Inoue et al. [53] where they demonstrated a variable expression pattern of epithelial mesenchymal transition (EMT) marker E-cadherin within female genital tissues from benign and malignant tumors compared to normal ovaries substantiates the variability factor in present study results. Precise gene expression profiles of individual cells comprising of tumor tissue may be quite variable and hence representing differential levels of expression and hence collectively all tumor cells contribute to heterogeneous cell types and thus variable representation of gene expression.

The CSC hypothesis proposes bi-directional inter-conversion between stem and non-stem compartments in order to sustain tumorigenesis and establish multi-lineage differentiation into different tumor cell types [54]. However, exact mechanism to explain such lineage differentiation remains unknown. OCT4^med/low^, CD44^hi^, CD24^+^ hierarchy of breast cancer cells have been reported recently [55]. Schwede et al. [56] characterized serous ovarian tumor cells, and co-related populations of stem cell like subtype in HG serous tumors to poor patient survival than the other differentiated subtype identified with mixed histology. Expression of stem cell markers (OCT4, SOX2, NANOG, NESTIN, ABCG2, CD133 and CD117) in ovarian cancer (sphere cells) represent their tumorigenic potential, and resistance to cisplatin, paclitaxel, adriamycin and methotrexate, which highlights the significance of “stemness” of cancer cells [17]. Similar to our current observations of two distinct populations of (spherical 5 μm and elliptical 10 μm) cells, recently two cell subsets with epithelial and stromal gene signatures respectively were detected in HG serous ovarian cancer exhibiting variable gene expression [57]. More recently, Ahmed et al. [58] based on proteomic signatures, reported activation/enrichment of specific pathways related to energy, metabolism and DNA repair in ascites derived tumor cells. Collectively these studies indicate persistence of heterogeneous CSC populations following a dynamic hierarchy possibly ordained to perform specific functions or cell rescue operations upon their exposure to onco-therapy. However, these reports fail to present a clear scenario of tumor progression associated phenotypic and genotypic changes in CSCs (specifically) in a stage-specific fashion. Recently, we reported presence of small spherical (~ 5 μm) and elliptical/spindle-shaped (≥ 10 μm) cells variably expressing CSC, and germline stem cell specific markers in normal ovarian and tumor tissues [10]. However, proliferation status of these (cancer) stem cell subsets especially in HG tumors (with high metastatic potential) remains unknown to date. To address this question, in our present study, we co-localized KI67 with stem and CSC specific markers and observed co-expression of KI67 and OCT4^+^, SSEA4^+^, CD44^+^, LGR5^+^, ALDH1/2^+^, CSCs, suggesting their highly proliferative nature.

### Co-expression studies for tumor biomarkers

Various CSC sub-populations viz. ALDH^+^, CD133^+^, CD44^+^/CD117^+^ presenting differential response to chemotherapeutic drug were recently isolated from ovarian cancer cell line SKOV3 [59]. Stemberger-Papic et al. [60] examined the expression of CD133^+^/CD117^+^ CSC markers in 64 serous ovarian cancer cases in HG serous carcinoma and peritoneal metastasis using IHC and found them relevant in disease progression and prognosis. Consistent with these investigators, presence of chemo-resistant CD44^+^/CD117^+^ CSCs from SKOV3 epithelial ovarian cancer cell line was reported by Chen et al. [61]. Similarly, Qin et al. [62] reported over-expression of Nestin unlike CD133, implicated in cisplatin resistance and shorter overall patient survival. In other cancer types dynamic and interconverting sub-populations of intestinal CSCs with LGR5^+^/KI67^+^, LGR5^+^/KI67^−^, LGR5^−^/KI67^+^, LGR5^−^/KI67^−^ expression profiles were reported in patient derived organoid xenografts generated in NOD/SCID mice [63], suggesting existence of various CSC sub-populations in patient tumors. Co-expressing populations of CD24^+^/CD44^+^/EpCAM^+^/CD133^+^ with pro-tumorigenic gene expression profile were reported in pancreatic ductal adenocarcinoma [64, 65]. In an independent study, Paula et al. [66] reported the presence of CD24^+^/CD44^+^/CD133^+^ CSCs in pancreatic ductal adenocarcinoma with higher expression of Notch1 and Sonic hedgehog. Expression of KI67 in these CSCs indicated tumor aggressiveness. In similar context, Paula et al. [66] reported ALDH1^+^/CD44^+^ co-expressing cells in non-malignant and neoplastic breast tissues. Similarly, Kalantari et al. [67] reported CD44^+^/CD133^+^ co-expressing putative CSC markers in prostate carcinoma. Consistent with these studies, expression of various stem and CSC-specific markers such as OCT4, SSEA4, ALDH, CD117, CD133, CD44, CD24, and LGR5 in our study, suggests presence of various CSC populations with characteristics which may explain heterogeneity, aggressiveness and metastatic potential of ovarian cancer. However, it remains unknown from our studies, if CSCs present in OSE layer migrate to ovarian cortex and progress to cancer or the stem cells locally present in the cortex region (or any migrating from extra-ovarian sites) undergo transformation and result into tumor development. The possible involvement of stem cells/CSCs from organs of extra-ovarian origin requires thorough and systematic investigation which may add novel dimension in understanding the complicated disease of ovarian cancer and explore the putative cells of origin. Tracking specific (cancer) stem cell sub populations across different tumor stages in comparison with normal ovary and further in-depth studies may provide greater clarity of this phenomenon. However, the choice of stem/CSC population (s) to chase their trafficking is interesting and will be part of our future study.

## Conclusions

To the best of our knowledge this study is the first attempt to investigate putative stem cell and CSC specific markers expressing and actively proliferating cell populations by dual labelling besides a stage-wise characterization of tumours compared to normal ovary focussing on OSE and cortex regions. Based on co-expression studies we found existence of dual positive proliferating stem cells and CSCs, few dormant stem/CSCs (SSEA4^+^/KI67^−^ and ALDH1/2^+^/KI67^−^) and only KI67^+^ cells signifying dynamic populations and interesting cellular hierarchy in the cortex region. It is only speculated that these dynamic cell populations/subsets are probably differentiating or propagating particular clone to serve specific function which was recently explained on the basis of guidance by micro environmental cues and selective pressures as per Darwinian theory [68]. However, this speculation with our study perspective requires further rigorous investigation.

Based on our studies, we conclude that various sub-populations of stem cells (in normal ovary) and CSCs (in ovarian tumor) exist in both OSE and cortex regions of the human ovary. We propose that stem cells present in the ovary may be undergoing transformation due to changes in microenvironment and cell signalling mechanisms and result into CSCs, which progress to tumor development and metastatic spread. This hypothesis needs to be tested and is under investigation in our laboratory. Our study thus offers a stepping-stone towards further quantitative evaluation of CSC sub-populations predominant at various tumor stages (especially metastatic stage) and associated mechanisms to aid early detection, diagnosis and devise apt therapeutic modalities to curb recurrence.

## Additional files

Additional file 1: **Figure S1.** Immunostaining for surface marker CD24 in sections of normal ovarian and tumor tissue sections: Mouse monoclonal anti-CD24 antibody was localized in normal ovarian and tumor tissue sections across NO, BN, BL, HG tissues in OSE (A, B) and ovarian cortex (C, D). Region between dotted boxes in A, C is magnified in B, D respectively. In (A, B) normal ovaries no staining was observed in OSE layer while (C, D) cortex shows only specific focal regions with multiple cells in cluster showing positive signals. In BN, BL and HG tissue, both OSE and cortex show positively stained cells but the extent of staining in cell membrane and moreover in the cytoplasm is more pronounced in BL and HG tumor samples. Cells marked in dotted squares are represented at higher magnification in insets. Additional insets in D of BN, BL and HG signify representative individual cell morphology, distribution density, localization and diverse staining pattern within the cortex. Scale bar = 100 μm (A, C) and 25 μm (B, D) respectively. (TIF 4223 kb)

Additional file 2: **Figure S2.** Immunofluorescence detection of ALDH1/2 in normal ovarian tissue and ovarian tumor sections: Spindle shaped ALDH1/2^+^ cells were observed in OSE layer (A) as well as cortex (B, C). HG OSE presents multi-layered ALDH1/2^+^ cells compared to NO, BN, BL OSE. NO, BN, BL cortex reveals elongated spindle shaped cells but those observed in HG cortex are moreover spherical and spindle-shaped with prominent ALDH1/2 signals. Clusters of ALDH1/2^+^ cells are typically observed in HG OSE and cortex both. Cells marked in dotted circles are represented at higher magnification in insets. White scale bar = 50 μm and blue scale bar = 10 μm (B, C). Alexa fluor 488 labelled secondary antibody was used and sections were counterstained with nucleus specific dye DAPI. (TIF 2264 kb)

Additional file 3: **Figure S3.** Immunohistochemistry of KI67 in normal ovarian tissue and tumor tissue sections: Monoclonal anti-KI67 antibody was localized and bright signals were acquired in both the OSE (A, B) and cortex (C, D) regions across NO, BN, BL and HG ovaries. Polar signals towards periphery in BN OSE layer (right inset) were observed while BL OSE displayed single bright KI67^+^ cells and signals throughout were nuclear with slight diffusion in the cytoplasm in certain cells. HG cortex displayed maximum number of KI67+ cells with nuclear signals and few membrane bound signals at periphery were also observed in individual KI67^+^ cells. Nuclei morphology varied as per cell cycle status of different proliferating cancer cells (including putative stem cells). Both elliptical and spherical nuclei were visible in all samples. NO, BN ovaries harboured relatively smaller sized cells compared to those in BL and HG cortex. Cells marked in dotted squares are represented at higher magnification in insets. Additional insets in B, D of NO, BN, BL, HG ovaries depict representative individual cell morphology, distribution density, localization and diverse staining pattern within the cortex. Scale bar = 100 μm (A, C) and 25 μm (B, D) respectively. (TIF 3954 kb)

Additional file 4: **Table S1.** Expression and distribution of markers within OSE and cortex regions of ovarian tissues by immunohistochemistry (IHC) method. (DOCX 20 kb)

Additional file 5: **Table S2.** Expression and distribution of markers within OSE and cortex regions of ovarian tissue by immunofluorescence (IF) method. (DOCX 19 kb)

Additional file 6: **Figure S4.** Negative controls for IHC and IF: Negative controls by omission of (anti-mouse and anti-rabbit) primary antibody with absent staining were documented by immunohistochemistry (A, B) and immunofluorescence (C, D) staining. OSE = ovarian surface epithelium, dotted lines in A, B denote OSE layer of cells in the section, Scale bar = 50 μm (C, D). (TIF 2121 kb)

## Abbreviations

BL: Borderline tumor
BN: Benign tumor
CSCs: Cancer stem cells
DAB: 3, 3′-diaminobenzidine
DAPI: 4′, 6-diamidino-2-phenylindole
EMT: Epithelial mesenchymal transition
hES: Human embryonic stem cells
HG: High grade tumor
IF: Immunofluorescence
IHC: Immuno-histochemistry
MSC: Mesenchymal stromal stem cell
NO: Normal ovary
OSE: Ovarian surface epithelium
PBS: Phosphate buffered saline
RT-PCR: Reverse transcription polymerase chain reaction
TAE: Tris-acetate-EDTA
VSELs: Very small embryonic like stem cells
