# Supplementary figures and images for: Characterization of stem cell and cancer stem cell populations in ovary and ovarian tumors
Source: J Ovarian Res. 2018 Aug 18;11:69. doi: 10.1186/s13048-018-0439-3 (PMC6098829; doi:10.1186/s13048-018-0439-3)

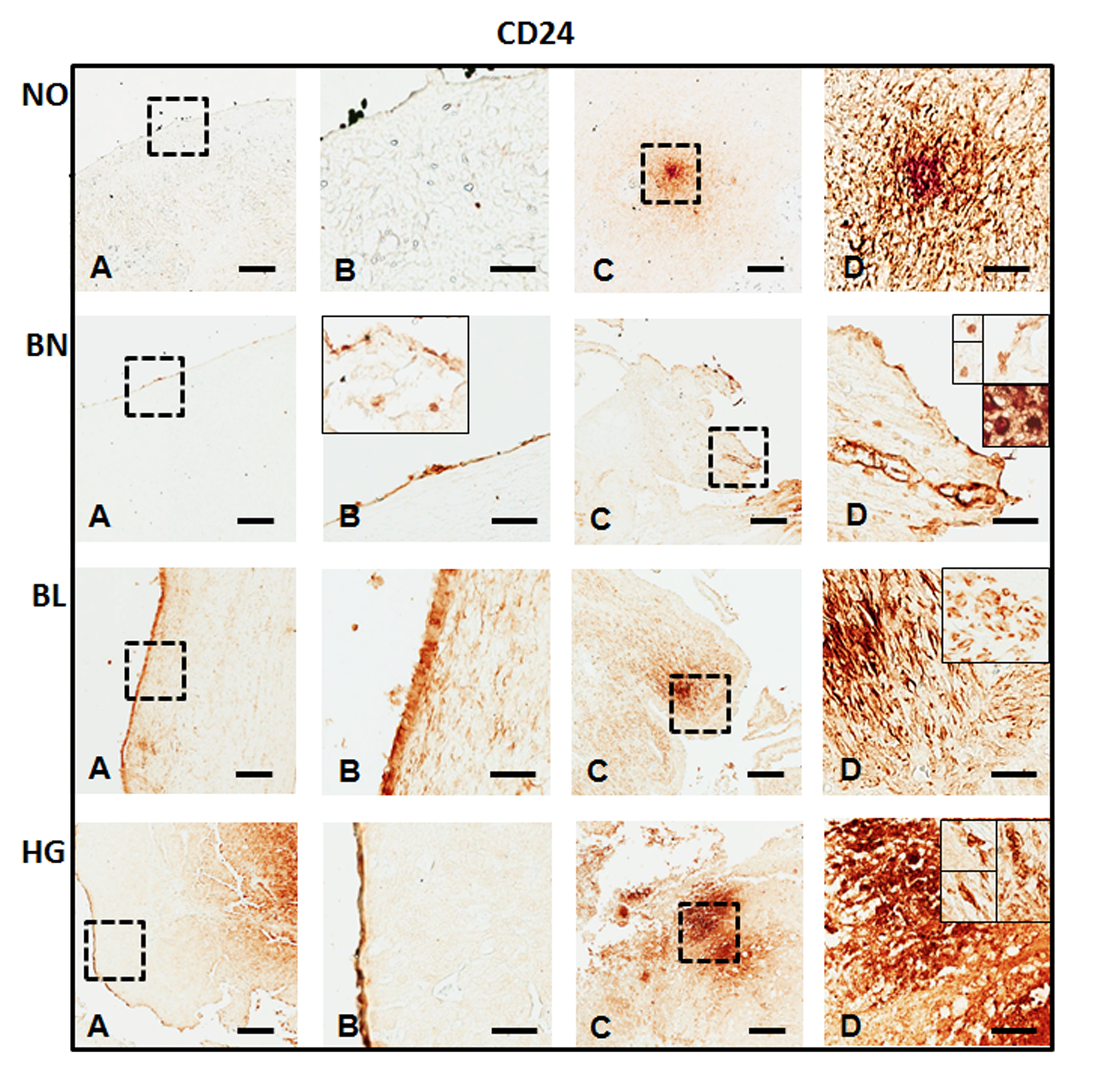

Supplement: Supplementary file 1 — Figure S1. Immunostaining for surface marker CD24 in sections of normal ovarian and tumor tissue sections: Mouse monoclonal anti-CD24 antibody was localized in normal ovarian and tumor tissue sections across NO, BN, BL, HG tissues in OSE (A, B) and ovarian cortex (C, D). Region between dotted boxes in A, C is magnified in B, D respectively. In (A, B) normal ovaries no staining was observed in OSE layer while (C, D) cortex shows only specific focal regions with multiple cells in cluster showing positive signals. In BN, BL and HG tissue, both OSE and cortex show positively stained cells but the extent of staining in cell membrane and moreover in the cytoplasm is more pronounced in BL and HG tumor samples. Cells marked in dotted squares are represented at higher magnification in insets. Additional insets in D of BN, BL and HG signify representative individual cell morphology, distribution density, localization and diverse staining pattern within the cortex. Scale bar = 100 μm (A, C) and 25 μm (B, D) respectively. (TIF 4223 kb) [file 13048_2018_439_MOESM1_ESM.tif]

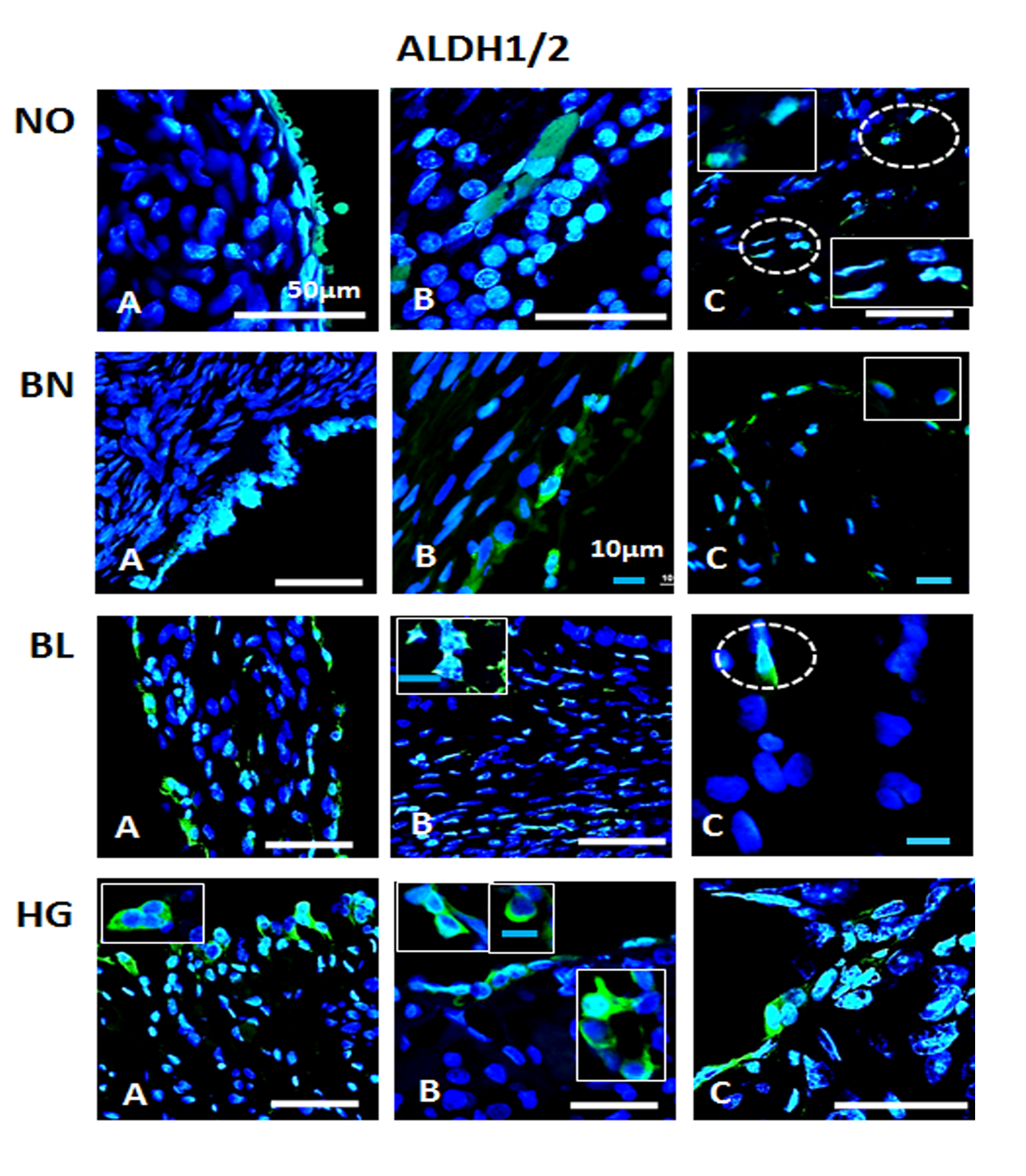

Supplement: Supplementary file 2 — Figure S2. Immunofluorescence detection of ALDH1/2 in normal ovarian tissue and ovarian tumor sections: Spindle shaped ALDH1/2+ cells were observed in OSE layer (A) as well as cortex (B, C). HG OSE presents multi-layered ALDH1/2+ cells compared to NO, BN, BL OSE. NO, BN, BL cortex reveals elongated spindle shaped cells but those observed in HG cortex are moreover spherical and spindle-shaped with prominent ALDH1/2 signals. Clusters of ALDH1/2+ cells are typically observed in HG OSE and cortex both. Cells marked in dotted circles are represented at higher magnification in insets. White scale bar = 50 μm and blue scale bar = 10 μm (B, C). Alexa fluor 488 labelled secondary antibody was used and sections were counterstained with nucleus specific dye DAPI. (TIF 2264 kb) [file 13048_2018_439_MOESM2_ESM.tif]

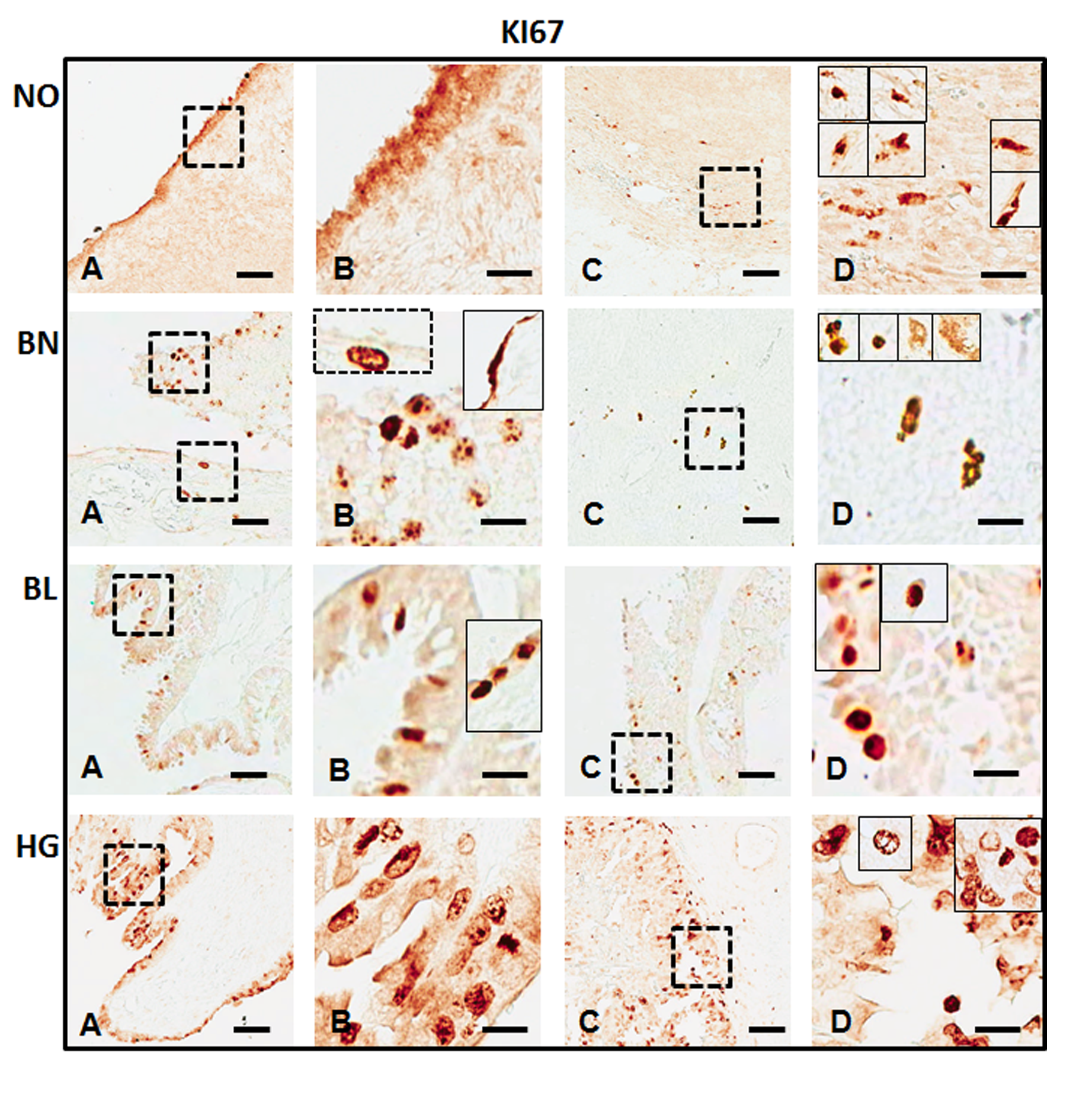

Supplement: Supplementary file 3 — Figure S3. Immunohistochemistry of KI67 in normal ovarian tissue and tumor tissue sections: Monoclonal anti-KI67 antibody was localized and bright signals were acquired in both the OSE (A, B) and cortex (C, D) regions across NO, BN, BL and HG ovaries. Polar signals towards periphery in BN OSE layer (right inset) were observed while BL OSE displayed single bright KI67+ cells and signals throughout were nuclear with slight diffusion in the cytoplasm in certain cells. HG cortex displayed maximum number of KI67+ cells with nuclear signals and few membrane bound signals at periphery were also observed in individual KI67+ cells. Nuclei morphology varied as per cell cycle status of different proliferating cancer cells (including putative stem cells). Both elliptical and spherical nuclei were visible in all samples. NO, BN ovaries harboured relatively smaller sized cells compared to those in BL and HG cortex. Cells marked in dotted squares are represented at higher magnification in insets. Additional insets in B, D of NO, BN, BL, HG ovaries depict representative individual cell morphology, distribution density, localization and diverse staining pattern within the cortex. Scale bar = 100 μm (A, C) and 25 μm (B, D) respectively. (TIF 3954 kb) [file 13048_2018_439_MOESM3_ESM.tif]

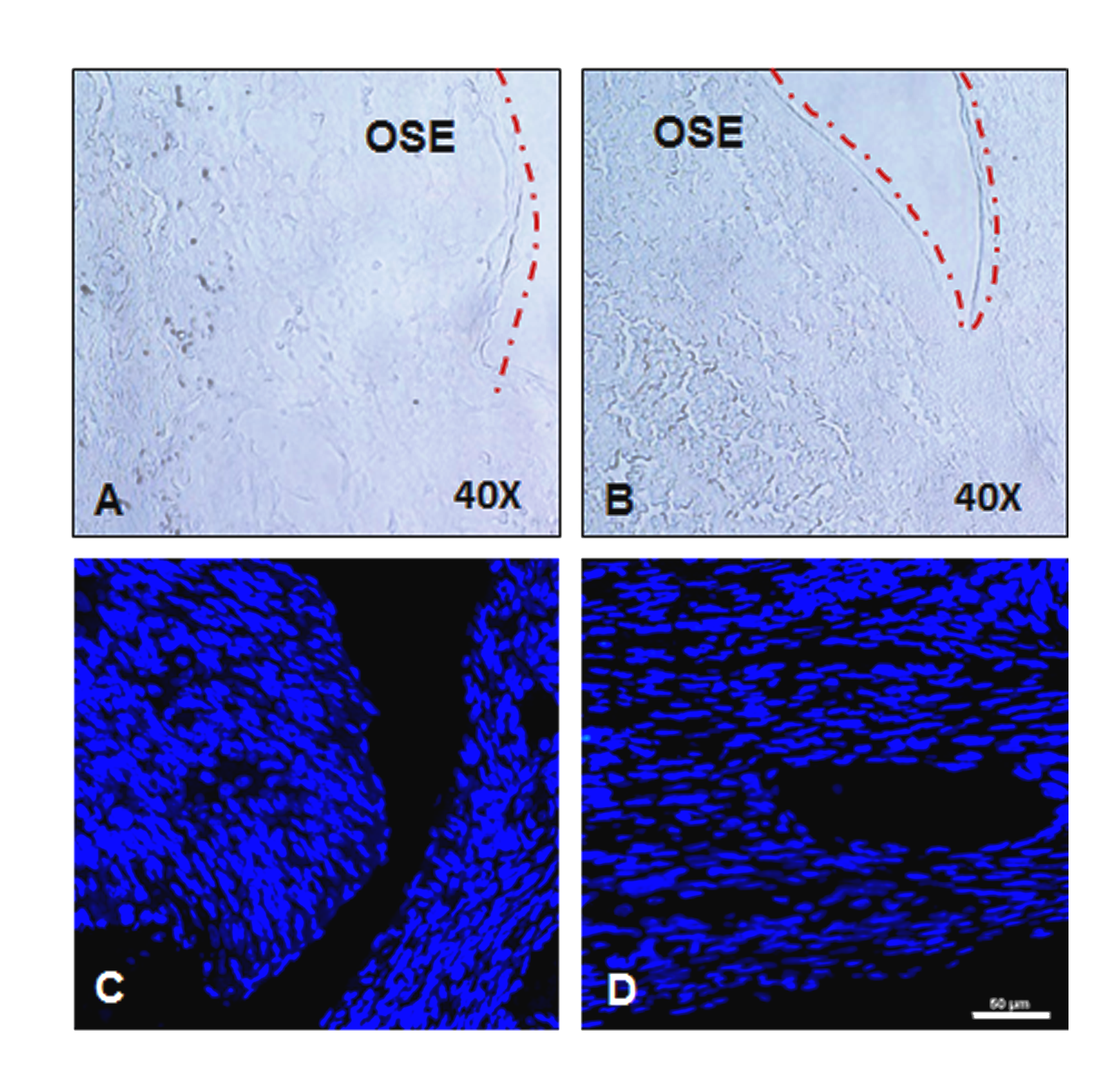

Supplement: Supplementary file 6 — Figure S4. Negative controls for IHC and IF: Negative controls by omission of (anti-mouse and anti-rabbit) primary antibody with absent staining were documented by immunohistochemistry (A, B) and immunofluorescence (C, D) staining. OSE = ovarian surface epithelium, dotted lines in A, B denote OSE layer of cells in the section, Scale bar = 50 μm (C, D). (TIF 2121 kb) [file 13048_2018_439_MOESM6_ESM.tif]
