# Supplementary material for: Characterization of stem cell and cancer stem cell populations in ovary and ovarian tumors
Source: J Ovarian Res. 2018 Aug 18;11:69. doi: 10.1186/s13048-018-0439-3 (PMC6098829; doi:10.1186/s13048-018-0439-3)
Supplement: Supplementary file 4 — Table S1. Expression and distribution of markers within OSE and cortex regions of ovarian tissues by immunohistochemistry (IHC) method. (DOCX 20 kb) [file 13048_2018_439_MOESM4_ESM.docx]

**Table S1: Expression and distribution of markers within OSE and cortex regions of ovarian tissues by immunohistochemistry (IHC) method**

| **Markers studied in various samples** | **Distribution of Markers** | |
| --- | --- | --- |
|  | **OSE** | **Cortex** |
| **C-KIT/CD117** | | |
| Normal Ovary | OSE cells & cell layer beneath stained +ve, no single isolated C-KIT+ cells observed | Specific regions with multiple cells in groups were identified as C-KIT+ rather than single isolated cells |
| Benign Tumor | Similar to NO, OSE layer showed specific + signals, few epithelial cells at periphery and few larger cells below OSE layer show bright signals | Single/cluster of + cells localized at many regions, specially oval shaped larger + cells, dim and bright signals in different cells within single field observed |
| Borderline Tumor | Larger columnar OSE cells with bright +ve signals diffused towards periphery | Few + cells in cluster had bright signals, larger specific signals with multiple + cells in cluster, very specific tiny spherical (<10µm) and larger elliptical cells (~10 µm) |
| High Grade Tumor | OSE cells revealed single isolated + cells and some multiple + OSE cells, round fluffy and elliptical single cells and few larger + cell clusters | Multiple regions with clusters of + cells with bright signals, single isolated spherical + (<10µm) cells |
| **CD133** | | |
| Normal Ovary | Specific + OSE cell layer with diffused/polar localization of signals at periphery | Single and multiple + cells spread in cortex region |
| Benign Tumor | Specifically stained + OSE cells | Single and multiple + cells with bright signals, some larger elliptical fluffy appearing cells in cluster |
| Borderline Tumor | Specific and polarized + signals in OSE cells towards periphery, multi-layered OSE cells show prominent signals | Very few and specific elliptical + cells spread throughout cortex |
| High Grade Tumor | Positive cells in OSE layer show diffused pattern | + cell clusters spread far away within cortex |
| **CD44** | | |
| Normal Ovary | Very few but specific + staining in OSE layer and in layer of cells beneath this layer | Multiple elliptical cells show + signals spread across cortex |
| Benign Tumor | Few single and multiple larger + cells embedded within OSE layer | Many single and elliptical cells in cluster, few fluffy appearing larger cells in groups/multiples show prominent + signals |
| Borderline Tumor | Few spherical and very specific + cells | Single isolated + cells were spread throughout in specific regions |
| High Grade Tumor | Single isolated and multiple bright + stained cells in cluster embedded in OSE layer and beneath | Many single and multiple bright + cells spread throughout cortex |
| **CD24** | | |
| Normal Ovary | No specific + signals but rare foci with faint staining in OSE layer | Few small foci with multiple + cells spread throughout cortex |
| Benign Tumor | Few OSE cells in a single layer revealed bright specific + signals | Tiny spherical single isolated cells or in multiples/groups with bright specific signals |
| Borderline Tumor | Single layer of OSE cells with + signals both bright and faint | Several isolated foci with multiple + cells at multiple regions throughout cortex, cytoplasmic and membrane bound localization was detected in CD24+ cells |
| High Grade Tumor | Single layer of OSE cells express bright + staining but single isolated/independent cells were not noticed | Several isolated foci with multiple + cells at multiple regions throughout cortex, cytoplasmic and membrane bound localization was detected in CD24+ cells |
| **KI67** | | |
| Normal Ovary | Specific + signals were detected in OSE layer and in some foci | Few but specific elliptical/spindle shaped + cells with nuclear and cytoplasmic signals |
| Benign Tumor | Very specific and bright signals with single isolated cells or in pairs/groups, elliptical cells with prominent cytoplasmic signals | Mitotic figures, punctate nuclear signals, bright nuclear and faint cytoplasmic signals were specifically observed in singles/multiples throughout |
| Borderline Tumor | Spherical and elliptical + cells with bright nuclear and faint cytoplasmic signals | Single isolated + cells with moreover nuclear and some cytoplasmic bright specific signals spread throughout |
| High Grade Tumor | OSE layer comprises of nuclear and cytoplasmic + cells, moreover elliptical shaped + cells detected | Small spherical and elliptical shaped multiple + cells with variable signals such as bright nuclear, punctate nuclear and cytoplasmic detected |

+: immuno-stain positive, OSE: Ovarian surface epithelium
