# Supplementary material for: Characterization of stem cell and cancer stem cell populations in ovary and ovarian tumors
Source: J Ovarian Res. 2018 Aug 18;11:69. doi: 10.1186/s13048-018-0439-3 (PMC6098829; doi:10.1186/s13048-018-0439-3)
Supplement: Supplementary file 5 — Table S2. Expression and distribution of markers within OSE and cortex regions of ovarian tissue by immunofluorescence (IF) method. (DOCX 19 kb) [file 13048_2018_439_MOESM5_ESM.docx]

**Table S2: Expression and distribution of markers within OSE and cortex regions of ovarian tissue by immunofluorescence (IF) method**

| **Markers studied in various samples** | **Distribution of Markers** | |
| --- | --- | --- |
|  | **OSE** | **Cortex** |
| **C-KIT/CD117** | | |
| Normal Ovary | Few specific OSE cells with bright + signals, or sometimes diffused signals at periphery | Multiple and few isolated single + (10 µm) cells visible |
| Benign Tumor | Single isolated and bright + signals in OSE | Single isolated or multiple + (≥10 µm) cells scattered throughout cortex |
| Borderline Tumor | Single layered columnar/ multi layered OSE cells reveal prominent signals | Single isolated as well as multiple cells in clusters reveal + signals |
| High Grade Tumor | Very specific single isolated and multiple + cell clusters | Very bright + tiny, specific and larger (>20 µm) cells |
| **CD133** | | |
| Normal Ovary | Single and multi-layered OSE with bright spindle shaped specific + OSE cells | Very bright + elliptical shaped single isolated cells |
| Benign Tumor | Very bright spindle/ elliptical shaped specific + OSE cells and layer beneath | Very bright + spherical and elliptical shaped single isolated (≥10 µm) cells |
| Borderline Tumor | Single and/or multi-layered OSE cells, few clusters with prominent and specific + cell signals | Single bright or small clusters with specific elliptical shaped + (≥20 µm) cells spread throughout |
| High Grade Tumor | Small spherical as well as elliptical shaped larger + cells in single/multi-layered OSE | Specific single isolated and multiple brightly stained spherical as well as elliptical shaped + cells (≤/≥10 µm) |
| **CD44** | | |
| Normal Ovary | Bright specific + cells in OSE layer and beneath with spherical and moreover elliptical morphology | Single isolated and multiple cells in cluster with elliptical morphology smaller and larger + cells (≥10 µm and ~20 µm) were spread throughout |
| Benign Tumor | Specific isolated single + cells embedded in OSE layer | Single isolated small spherical, large elliptical and other larger round fluffy appearing + cells (5-10-20 µm), few larger multi-nucleated clusters also detected |
| Borderline Tumor | Single layer of + OSE cells, single isolated cells and multi-nucleated clusters in vicinity of OSE layer | Few but specifically and bright stained + single isolated cells spread throughout of spherical and elliptical shapes (~10µm) |
| High Grade Tumor | Single/multi-layered OSE cells with bright + signals | Small spherical and large elliptical shaped + cells, moreover multi-nucleated larger structures/clusters (~20µm) spread throughout |
| **ALDH** | | |
| Normal Ovary | Single/multi-layered + OSE cells with spherical and elliptical/spindle shape | Moreover elliptical shaped (≥10/~25 µm) and multiple isolated spherical + cells present |
| Benign Tumor | Similar to NO, (≥10µm) elliptical/spindle shape + cells embedded in OSE layer | Elliptical shaped (≥10 µm) + cells either single isolated or multiples spread throughout |
| Borderline Tumor | Few but specific and bright stained + OSE cells of both spherical and elliptical/spindle morphology | Elliptical shaped single isolated + cells (~10µm) spread throughout |
| High Grade Tumor | Spherical and elliptical/spindle shape + OSE cells, multi-nucleated clusters and multiple + cells in layer beneath | Single isolated, spherical and elongated / elliptical shaped, few clusters of + cells (~10 µm) cells |

+: immuno-stain positive, OSE: Ovarian surface epithelium
